# *Escherichia coli* DNA replication: the old model organism still holds many surprises

**DOI:** 10.1093/femsre/fuae018

**Published:** 2024-07-09

**Authors:** Krystian Łazowski, Roger Woodgate, Iwona J Fijalkowska

**Affiliations:** Laboratory of DNA Replication and Genome Stability, Institute of Biochemistry and Biophysics, Polish Academy of Sciences, Pawinskiego 5a, 02-106 Warsaw, Poland; Laboratory of Genomic Integrity, National Institute of Child Health and Human Development, National Institutes of Health, Bethesda, MD 20892-3371, United States; Laboratory of DNA Replication and Genome Stability, Institute of Biochemistry and Biophysics, Polish Academy of Sciences, Pawinskiego 5a, 02-106 Warsaw, Poland

**Keywords:** *Escherichia coli* replisome, DNA polymerase III holoenzyme, DNA replication fidelity, ribonucleotide excision repair, single-molecule live-cell imaging, Cryo-EM

## Abstract

Research on *Escherichia coli* DNA replication paved the groundwork for many breakthrough discoveries with important implications for our understanding of human molecular biology, due to the high level of conservation of key molecular processes involved. To this day, it attracts a lot of attention, partially by virtue of being an important model organism, but also because the understanding of factors influencing replication fidelity might be important for studies on the emergence of antibiotic resistance. Importantly, the wide access to high-resolution single-molecule and live-cell imaging, whole genome sequencing, and cryo-electron microscopy techniques, which were greatly popularized in the last decade, allows us to revisit certain assumptions about the replisomes and offers very detailed insight into how they work. For many parts of the replisome, step-by-step mechanisms have been reconstituted, and some new players identified. This review summarizes the latest developments in the area, focusing on (a) the structure of the replisome and mechanisms of action of its components, (b) organization of replisome transactions and repair, (c) replisome dynamics, and (d) factors influencing the base and sugar fidelity of DNA synthesis.

## Teaser

This review provides an updated view on the *Escherichia coli* replication fork, in particular its structure, dynamics, and factors influencing DNA replication fidelity, based on the latest discoveries enabled by genetic and biochemical approaches, cryo-electron microscopy, live-cell imaging, and whole genome sequencing.

## Introduction

It has been over half a century since the discovery of the first DNA polymerase that Kornberg and colleagues isolated from the commensal bacterium *Escherichia coli* in 1956 (Lehman et al. 1958). Nowadays, *E. coli* is a well-established model organism for studies of DNA synthesis. Its use facilitated many pioneering works that became the cornerstone of the recognized model of DNA replication, including the semiconservative model of genome duplication described by Meselson and Stahl in their classic paper from 1958 (Meselson and Stahl 1958), the discontinuous mechanism of lagging-strand synthesis proposed by Okazaki in 1971 (Okazaki et al. 1971), but also the very nature of mutations (Luria and Delbrück 1943, Cairns et al. 1988). These findings are generally universal for all domains of life. Still, more recent discoveries, enabled in particular by the development of high-resolution single-molecule imaging, whole genome sequencing (WGS), and cryo-electron microscopy (Cryo-EM), paint a much more complex picture of *E. coli* DNA replication than initially thought. This review aims to summarize the available knowledge about *E. coli* DNA replication with particular interest in the developments of the last decade.

## DNA replication by the replisome

DNA replication is a highly evolutionarily conserved process that requires a coordinated action of multiple proteins responsible for the timely and accurate execution of different tasks that can be grouped into three stages: initiation, elongation, and termination (Yao and O’Donnell 2016). Bacterial genomes, which are frequently small, circular chromosomes (such as the ∼4.6 Mb genome of *E. coli*), have their replication initiated from a single defined origin site (*oriC*) containing DnaA-binding boxes recognized by the DNA replication initiator protein DnaA (Kaguni 2011, Trojanowski et al. 2018). Unlike eukaryotic cells, where origins are licensed for DNA replication well before the S-phase of the cell cycle (when DNA is replicated), in many bacteria, replication may be initiated several times before cell division. As a consequence, daughter cells may inherit genomes that already undergo another cycle of replication (the so-called “multifork replication”) (Fossum et al. 2007).

Cooperative binding of the DnaA molecules at *oriC* promotes the unwinding of an A:T-rich DNA fragment and loading of the DnaB_6_–DnaC_6_ complexes onto each exposed single-stranded DNA fragment. The binding of the DnaG primase to the DnaB helicase with DnaC dissociation allows for replisome assembly, completing the last major step of DNA replication initiation (reviewed in Katayama 2017). DnaG primase synthesizes ∼10-nt long RNA primers removed at a later step (Zechner et al. 1992a). Note that in the simplified model of *E. coli* DNA replication initiation presented above, only the primary proteins involved were described. A more comprehensive view on the bacterial replication initiation factors can be found in Grimwade and Leonard (2021).

The elongation stage of DNA replication is carried out by multiple proteins constituting functional complexes called replisomes. The characteristics of the *E. coli* replisome, which is the focus of this review, will be described in the following sections.

The last step of DNA replication is termination, which happens when the two replisomes approaching from opposite directions converge. *E. coli* replication termination system is centered around the so-called termination region containing 10 *terA*–*J* sites that interact with Tus proteins. These sites are oriented in such a way that the replication machinery bypasses the first five, but is trapped at one of the remaining five, which in turn are easily bypassed by the replisome approaching from the opposite direction. Consequently, both replisomes become trapped in the termination region to avoid chromosome over replication and give the cell time to process replication intermediates (Rudolph et al. 2009, 2013). Termination is observed predominantly at the four innermost sites, *terA*–*terD* (Ivanova et al. 2015, Dimude et al. 2018). Interestingly, replisome trapping might be a source of genome instability as recent work utilizing next-generation-based methods [X-seq and END-seq that map the chromosomal positions of Holliday junctions (HJs) and double-stranded breaks (DSBs), respectively] identified *ter* sites as the region of frequently occurring HJs and DSBs (Mei et al. 2021). According to the proposed model, these may arise as a consequence of replication fork stalling and the arrival of another codirectional replisome before fork convergence, resulting in the displacement of the leading strand, DSB end resection, and involvement of homologous recombination (HR) machinery that generates HJs. However, these one-ended DSBs cannot be repaired until the sister replisome arrives, leading to the accumulation of repair intermediates (Mei et al. 2021). DNA replication termination was reviewed recently in Goodall et al. (2023).

### *Escherichia coli* replisome organization

The DNA elongation step is carried out by the replisomes (reviewed in Yao and O’Donnell 2016, Xu and Dixon 2018). The two most essential tasks for the replisome are (1) separation and (2) semiconservative duplication of the two parental DNA strands. More recent evidence suggests that replisomes also play a role as sensors of obstacles that hinder replication progression and facilitate their repair and tolerance via different interactions (Hawkins et al. 2019, Wolak et al. 2020, Thrall et al. 2022). The two major models that explain the spatiotemporal organization of replication describe the replisomes as either mobile complexes that run along DNA like a train on a track or stationary factories anchored at a certain location in the cell with DNA pushed through. In bacterial research, the dominant view is that the replisomes are fixed in space, supporting the factory model (Lemon and Grossman 1998, Brendler et al. 2000, Mangiameli et al. 2018). However, both modes were directly observed using live cell imaging in slow-growing *E. coli* (Bates and Kleckner 2005, Reyes-Lamothe et al. 2008, Mangiameli et al. 2017, Japaridze et al. 2020), leading to the hypothesis that at the beginning of DNA replication, the two replisomes are cohered but at some point before termination the two sister replisomes may break apart and travel separately, at least under slow-growth conditions. Recent evidence suggests that the early cohesion of sister replisomes facilitates the establishment of the replication fork and its successful progression, while loss of this interaction increases replication fork stalling and the involvement of replication restart protein RecB (Chen et al. 2023). The cohesion of the replisomes might, for example, help with the coordination of both replication forks for timely cohesion at the termination site by slowing down one replicative machinery when the other deals with obstacles such as transcription complexes, which are particularly abundant at the early replicating region, known to be heavily transcribed in *E. coli* [see the section “Discussion” in Chen et al. (2023)].

**Figure 1.**
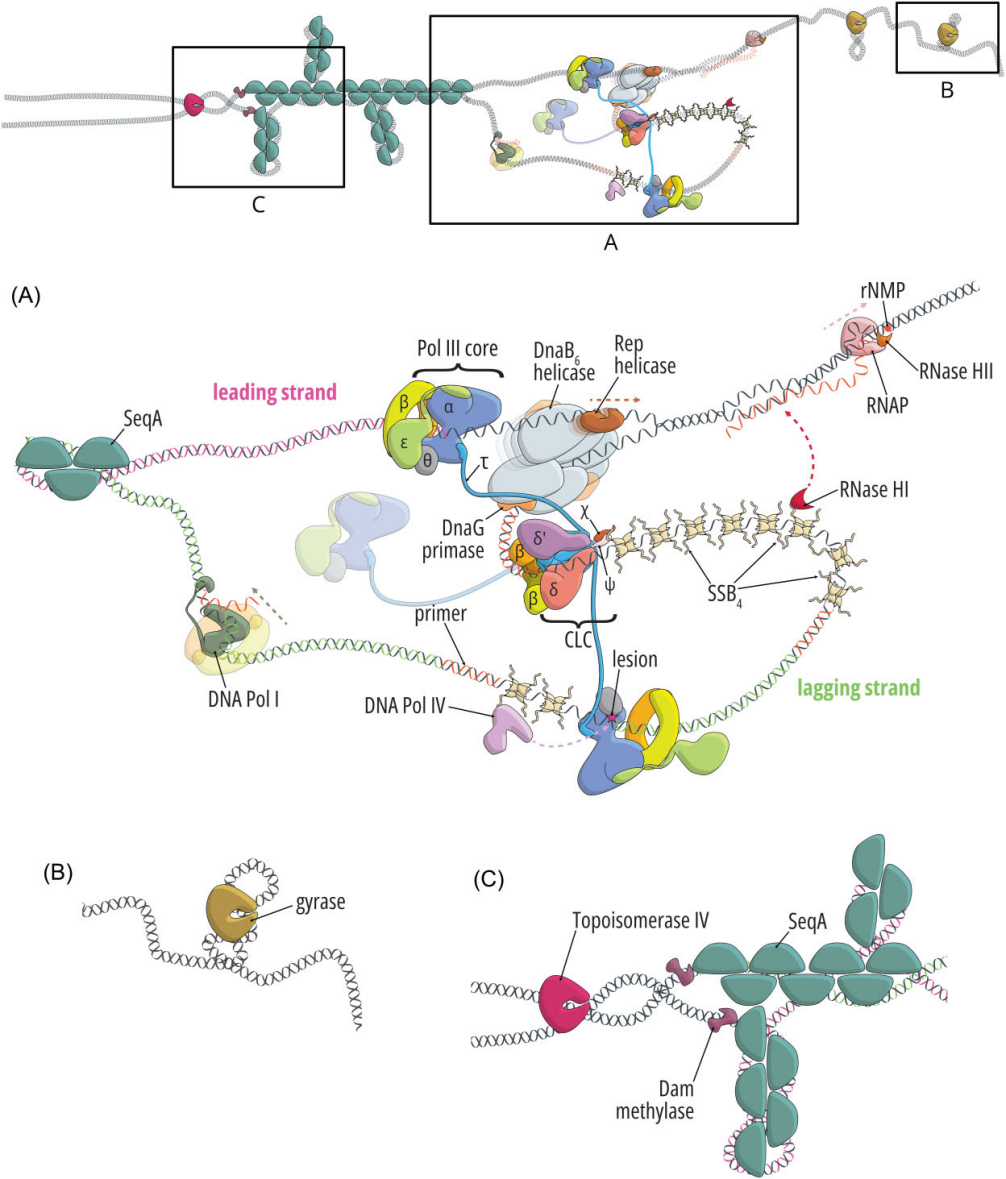
*Escherichia coli* replication fork and its surroundings. The replisome (A) and the major sites of activity in front of (B) and behind (C) the fork are magnified. (A): The multisubunit replisome consists of the DnaB helicase, the DnaG primase, tetrameric SSB proteins, the CLC, and 2–3 identical Pol III cores responsible for replication. These subassemblies are interconnected by a plethora of interactions. However, more recent evidence shows that replisome makes contact with many other proteins, so as to enrich and recruit them to the site of DNA synthesis. The purpose of these interactions is to facilitate DNA damage repair or tolerance (e.g. an interaction between the C-terminal tail of SSB with DNA Pol IV is shown in the picture), Okazaki fragment maturation (DNA Pol I), or removal of frequently encountered replication obstacles such as proteins (interaction between the DnaB helicase and the Rep helicase) and RNA transcripts (interaction between the C-terminal SSB tail and RNase HI). However, the mechanism of recruitment of some of them (e.g. mismatch repair proteins or Pol I) remains to be uncovered. (B): Movement of the replication fork increases the topological stress related to the accumulation of positive supercoils, which in front of the fork are relaxed by the gyrase. (C): The supercoils may migrate behind the fork by virtue of replisome rotation, leading to the entanglement of sister chromosomes, which is resolved predominantly by topoisomerase IV. Topo IV is temporally and spatially separated from the ongoing replication by the SeqA protein filaments that protect the immediate vicinity of the replisome, delaying DNA disentanglement and methylation.

In *E. coli*, the single replicative polymerase, or the replicase, DNA polymerase III holoenzyme (Pol III HE), is responsible for the lion’s share of DNA replication. Pol III HE is a complex of 10 distinct proteins that can be organized into three subassemblies: the polymerase core (Pol III), the sliding clamp, and the clamp loader complex (CLC; Fig. 1A) (McHenry 2011, Yao and O’Donnell 2016). An important integral part of the replisome is the helicase DnaB_6_. There is also a plethora of proteins that associate with the replisome either transiently or for an extended period of time, such as the primase DnaG, accessory DNA polymerases, the single-stranded-DNA-binding (SSB) proteins, topoisomerases, or some repair proteins such as RNase HI. These will be discussed in the following parts of this review.

#### The DnaB helicase

The major *E. coli* replicative helicase is a homohexamer of DnaB subunits encoded by the *dnaB* gene (reviewed in Lewis et al. 2016, Xu and Dixon 2018). DnaB_6_ translocates 5′→3′ on the lagging-strand template (Fig. 1A). DnaB is loaded onto single-stranded DNA (ssDNA) as a part of the DnaB_6_–DnaC_6_ complex, with the help of DnaA at *oriC* at the very beginning of DNA replication [see Blaine et al. (2023) for a review]. Recent crystal structures of the DnaB_6_–DnaC_6_ complex suggest that DnaC binding via its NTD to the CTD of DnaB causes a distortion in the helicase ring. This distortion accumulates when more DnaC units are bound and eventually results in the helicase opening (Chodavarapu et al. 2016, Chase et al. 2018, Arias-Palomo et al. 2019, Nagata et al. 2020). ATP binding in the DnaC ATPase domain stabilizes DnaB open conformation and is important for helicase activation (Arias-Palomo et al. 2019, Puri et al. 2021). During loading, DnaB assumes a dilated conformation with a wide central channel, but it can also dynamically switch between dilated and constricted conformations as it travels on the ssDNA (Strycharska et al. 2013).

The primary role of the helicase is to unwind the DNA duplex and produce single-stranded fragments, which is powered by ATP hydrolysis. The ATP-binding sites positioned apically at the replication fork are located in the C-terminal domains (CTDs), whereas the N-terminal domains (NTDs) that form a trimer of dimers participate in protein–protein interactions with various partners. The two are connected via linker domains. The initial insight into the mechanism of translocation was provided thanks to studies involving viral and eukaryotic models (Enemark and Joshua-Tor 2006, also reviewed in Li and O’Donnell 2018), and the so-called hand-on-hand model that resembles rope climbing has been proposed for DnaB based on the crystal structure (Itsathitphaisarn et al. 2012). According to this model, during translocation, the helicase assumes a spiral staircase-like conformation around ssDNA and binds 12 nucleotides (2 per subunit). ATP hydrolysis drives the movement of the CTD of the 5′-most DnaB subunit toward the 3′-most subunit, resulting in the unwinding of 2 nucleotides on the lagging strand. For every two movements of the CTDs, the dimeric NTDs would move. The hexameric state of the helicase would be maintained by the presence of flexible linkers. Neither DNA nor the helicase itself rotates during translocation (Itsathitphaisarn et al. 2012). The helicase alone *in vitro* unwinds at the rate of around 30–35 nt s^–1^ (Kim et al. 1996) but is greatly stimulated by the replisome subunits to support the *in vivo* rates of replisome progression (Chandler et al. 1975, Mok and Marians 1987). Interestingly, single-molecule FRET studies suggest that the external surface of DnaB interacts with the excluded strand, downregulating its progression rate (Carney et al. 2017).

Another role of the helicase is a structural one. *In vitro* single-molecule fluorescence microscopy studies revealed that DnaB is the most stable component of the replisome, as upon binding it stays associated for up to 30 minutes (Beattie et al. 2017, Spinks et al. 2021). In principle, such a long dwell time allows it to remain bound at the replication fork for the whole period of DNA replication. Based on these observations, it has been proposed that DnaB might serve as the anchor that not only organizes replisome assembly but also enables its dynamic and stochastic nature, which has become evident in recent years (Beattie et al. 2017). DnaB interacts with DnaA and DnaC, as well as the DnaG primase and the τ subunit of the replicase, anchoring both to the replisome. Recent work utilizing genetic and live cell imaging approaches suggests frequent interactions between DnaB and the Rep helicase at the replication fork *in vivo*, allowing for enrichment of the repair helicase near the sites of DNA synthesis, likely to aid barrier removal in front of the fork, which is supported by *in vitro* data (Fig. 1A) (Brüning et al. 2018, Syeda et al. 2019). Another important interacting partner is PriC, a protein involved in replisome reassembly and replication restart (Wessel et al. 2013, 2016).

What happens to the helicase near the termination sites is not well understood. In principle, the helicase could displace the 3′ end of the newly synthesized leading strand from the opposite fork and continue unwinding. However, biochemical studies suggest that in this scenario, DnaB rather encircles double-stranded DNA (dsDNA), which would preclude any unwinding (Kaplan and O’Donnell 2002). Encircling both DNA strands requires DnaB to be in a nonconstricted state when it is known to unwind more slowly (Strycharska et al. 2013). Additionally, DnaC was shown to facilitate helicase unloading in an ATP-dependent manner (Puri et al. 2021). It is, therefore, possible that slowing down the helicase somehow allows it to bind DnaC, which aids its detachment from DNA. Another possibility is that perhaps the two helicase hexamers cannot pass each other and collide head-on, essentially blocking further translocation. Which model persists and what exactly could be the signal for this remains to be understood.

#### The DnaG primase

The monomeric primase is the product of the *dnaG* gene (Rowen and Kornberg 1978). DnaG synthesizes 10–12-nt long RNA primers that start DNA replication (Fig. 1A) (Kitani et al. 1985): at least one that primes the leading strand and roughly 2000 that prime each Okazaki fragment on the lagging strand. RNA synthesis in *E. coli* starts at template dCTG sequences (Swart and Griep 1995).

DnaG contains three important regions: the zinc-binding domain (ZBD) located at the N terminus, the RNA polymerase domain (RPD), and the CTD (DnaGC) responsible for interaction with the helicase. DnaGC interacts with DnaB NTD, albeit weakly, and this interaction is important to support *in vivo* rates of RNA synthesis (Johnson et al. 2000, Mitkova et al. 2003, Manosas et al. 2009), as DnaG on its own binds DNA weakly and is not very efficient (Khopde et al. 2002, Corn et al. 2005). This interaction starts during DNA replication initiation when synthesis of the first primer promotes subsequent association of the replicase. Based upon biochemical studies, the presumed stoichiometry was 1 DnaB hexamer to 2–3 DnaG monomers (Mitkova et al. 2003), but structural and biochemical studies suggest that during processive DNA replication, it might actually be 1:1 (Itsathitphaisarn et al. 2012), especially given that multiple DnaG monomers bound to the helicase seem to have an inhibitory effect on the replisome (Tanner et al. 2008). In the same structural analysis, Itsathitphaisarn et al. (2012) put forward the hypothesis that the contact between DnaB and DnaG might not necessarily be maintained for the whole period of primer synthesis. As DnaG can be bound to the lagging DNA strand via the interaction with SSB (Yuzhakov et al. 1999), it is thus possible that the transient DnaB–DnaG interaction is more important for the deposition of the primase on the template strand and/or quick termination of priming reaction on the one hand, and for the dynamic control of the behavior of DnaB on the other hand, which will be discussed later. It has been suggested that two DnaG copies acting *in trans* are required for primer synthesis, with one responsible for template recognition via the ZBD and the other carrying out primer synthesis using RPD (Corn et al. 2005).

After primer synthesis, DnaG remains bound to the primer and needs to be displaced by the χ subunit of the CLC of the replicase to allow for loading of the processivity factor (Fig. 1A) (Yuzhakov et al. 1999, Manosas et al. 2009). This finalizes the cycle of DnaG in the replisome.

#### The CLC

The CLC is composed of seven subunits (δδ′τ_(2/3)_γ_(1/0)_ψχ) and plays multiple roles in the replisome. These functions are brought together via interactions with the five domains (I–V) of the τ subunit. The τ subunit Domain V interacts with the Pol III core—there are 2–3 cores (described later) responsible for DNA synthesis and proofreading (Fig. 1A). One core replicates the leading strand, while the other one or two replicate the ∼1000-nt long Okazaki fragments that together constitute the lagging strand (Okazaki et al. 1971, Zechner et al. 1992b, Tougu and Marians 1996).

It was initially assumed that there are two cores that simultaneously replicate both leading and lagging DNA strands. Under this scenario, there are two τ subunits, while the third is replaced by its shorter variant (γ subunit) truncated at the C end, and thus lacking the core-interacting and the helicase-interacting Domains IV and V, respectively. While both τ and γ subunits are products of the *dnaX* gene, the shorter γ subunit is a consequence of ribosomal frameshifting taking place during translation (Blinkowa and Walker 1990). However, an *in vitro* study showed that a tri-core replicase could be functional in the replisome (McInerney et al. 2007). Soon thereafter, in a series of experiments utilizing fluorescently tagged replisome subunits, it was shown that the *in vivo* stoichiometry of the CLC observed in live-cell imaging is τ_3_δδ′(αεθ)_3_ψχ (Reyes-Lamothe et al. 2010). It has been proposed that the purpose of the third τ-bound core might be to facilitate simultaneous replication of two Okazaki fragments (McInerney et al. 2007, Montón Silva et al. 2015), but another explanation is that only two cores occupy the sliding clamps, while the third might wait for another clamp to be loaded (Reyes-Lamothe et al. 2010). Additionally, single-molecule studies showed that the tri-core replicase exhibits higher processivity than the two-core isoform (Georgescu et al. 2012). This might be related to the fact that Domain IV was also shown to interact with DNA (Jergic et al. 2007). Nevertheless, a body of data supports the idea that the replisome might contain only two cores, at least under certain conditions (also discussed in McHenry 2011). While the γ subunit is not essential for survival (Blinkova et al. 1993), it copurifies within the replisome (McHenry 1982, Glover and McHenry 2001, Dohrmann et al. 2016) and is also present in other bacteria (Larsen et al. 2000, Tashjian and Chien 2023). Additionally, cells lacking the γ subunit show increased UV-sensitivity and defective DNA synthesis by one of the translesion synthesis (TLS) polymerases, DNA polymerase IV (Pol IV), suggesting impaired DNA repair and/or damage tolerance (Dohrmann et al. 2016). Consistent results were also obtained in another bacterium, *Caulobacter crescentus* (Tashjian and Chien 2023). One possibility is that the third core outcompetes Pol IV under stress conditions, causing impaired damage response, but also during the stationary phase, leading to selective disadvantage as low-fidelity polymerases are strongly expressed during that time and play a role in adaptation (Yeiser et al. 2002, Corzett et al. 2013). On the other hand, a three-core replicase might be handy under nutrient-rich conditions, increasing the processivity of DNA synthesis during fast growth. This suggests that perhaps replicase composition is regulated depending on the cellular state.

The τ subunit Domain IV interacts with the DnaB helicase (Fig. 1A). The exact interface is not known but is presumed to be dynamic. This interaction is important for processive DNA replication and possibly for sensing the uncoupling of DNA unwinding and synthesis, as the helicase translocation rate depends on τ binding (Kim et al. 1996, Graham et al. 2017). Newly obtained biochemical data suggest that a single copy of the τ subunit participates in the DnaB binding (Monachino et al. 2020). The strength of this interaction increases when DnaG is also bound to the helicase (Monachino et al. 2020). A textbook view of this interaction was that it remains stable for extended periods of time during replication extension; however, single-molecule studies suggest that this is not the case (discussed later) (Lewis et al. 2017).

The τ subunit Domains I–III participate in the key CLC activity, which is loading the β_2_ clamp onto DNA (Fig. 1A). In particular, Domains I and II form an AAA+ interface and exhibit ATPase activity, whereas Domain III is the collar domain that comes into contact with the ψ subunit (Simonetta et al. 2009). Domains I–III are common for τ, δ, and δ′, although δ and δ′ do not provide the ATPase activity and do not interact with ψ. Together, they form a pentameric structure in which Domain I create a C-shaped passage for DNA and contact with the clamp, while Domain III form a ring-like collar that sits atop the other domains (Simonetta et al. 2009).

The subunits are ordered as follows: δ, γ/τ_1_, γ/τ_2_, γ/τ_3_, and δ′ (Kazmirski et al. 2004). Recently, a series of Cryo-EM structures of the CLC in various conformational states and quaternary complexes with the β_2_ clamp and/or DNA have been published, offering insight into the clamp loading cycle (Xu et al. 2023). The first step is binding three ATP molecules at the interfaces connecting γ/τ subunits, as well as between γ/τ and δ′. ATP binding induces conformational changes, leading to the reorganization of the AAA+ domains (Hingorani and O’Donnell 1998, Ason et al. 2003). This makes the CLC competent in binding the β_2_ clamp, which is the next step of the cycle. Biochemical analysis of the β_2_ mutants with a destabilized dimer interface as well as fluorescence proximity sensing assay suggest that the ATP-bound CLC actively opens closed clamps rather than simply capturing and stabilizing open clamps from the cytosol (Paschall et al. 2011, Douma et al. 2017). β_2_ binds first via the δ subunit. The gap between δ and δ′ Domains I then expands through a crab claw-like movement, leading to the opening of β_2_ (Xu et al. 2023).

As the next step, a primer–template DNA duplex passes through the open channel formed between δ and δ′ subunits of the pentameric ring (Tondnevis et al. 2016). Interestingly, the crystal structure of the CLC in complex with DNA suggests that virtually only the template strand is in contact with the CLC (Simonetta et al. 2009). The downstream part of the template strand exits through the gap between Domains I, II, and III of the δ subunit, where the highly conserved loop at positions 276–283 on the exterior surface of the δ collar domain establishes an interaction with the template strand (Chen et al. 2008). The exterior surface is positively charged and seems to be interacting not only with the template DNA but also with the downstream part of the gapped or nicked nascent strand, providing a structural basis for how the CLC loads the β_2_ clamps on such duplexes (Xu et al. 2023). Although the structures do not show the position of the highly flexible C-terminal part of the τ subunit, it is known that Domain IV weakly binds ssDNA and dsDNA (Jergic et al. 2007), and thus, it cannot be excluded that this interaction might further stabilize DNA around the CLC.

DNA binding prompts a conformational change with the tightening of the AAA+ interface, bringing the arginine fingers close to the ATP molecules and facilitating their concerted hydrolysis (Xu et al. 2023). This triggers the release of the clamp-encircled DNA from the CLC.

The τ/γ subunit is also in contact with the ψχ tail of the CLC. The two homologs χ and ψ are the products of *holC* and *holD* genes, respectively. They form a dimer with a highly conserved interaction interface, and neither contacts DNA directly (Gulbis et al. 2004). A SAXS structure of the seven-subunit CLC suggests that the ψχ dimer is located close to γ/τ_3_ (Tondnevis et al. 2015). The N-terminal part of the ψ subunit penetrates the collar domain, interacting with the three τ/γ subunits (Simonetta et al. 2009). This not only increases the strength of interactions within the pentamer complex (Olson et al. 1995), but also helps the CLC to assume the conformation favored during DNA binding, increasing the affinity by 20-fold (Simonetta et al. 2009). The χ subunit interacts with the SSB proteins coating the exposed single-stranded regions of the template DNA strand (Marceau et al. 2011) and participates in their remodeling, as suggested based on *in vitro* FRET assays (Newcomb et al. 2022). The interaction of the CLC with SSB stabilizes the complex on primer–template DNA (Newcomb et al. 2022, Xu et al. 2023) and is also important for processive Okazaki fragment synthesis (Fig. 1A) (Glover and McHenry 1998). The multiple roles of SSB in the replication fork will be discussed later.

#### The β_2_ clamp

The β_2_ clamp is a ring-shaped homodimer encircling the primer–template duplex. The clamp is a homodimer consisting of two 41-kDa proteins encoded by the *dnaN* gene (Burgers et al. 1981). The primary purpose of the β_2_ clamp in the replication fork is to increase the speed and the processivity of DNA replication. This is best illustrated by the *in vitro* biochemical activity of the Pol III core, which alone replicates ∼20 nt s^–1^ and 10–20 nt per binding event (Fay et al. 1981, Maki and Kornberg 1985). These numbers jump to ∼350–500 nt s^–1^ and up to ∼2000 nts per binding event when bound to the β_2_ clamp (Tanner et al. 2008), and to ∼700–1000 nt s^–1^ and ∼150 000 nts per binding event in the context of the replisome (Mok and Marians 1987, Yao et al. 2009, Tanner et al. 2011). All *E. coli* DNA polymerases were shown to increase their processivities 25–400 times upon β_2_ binding.

**Figure 2.**
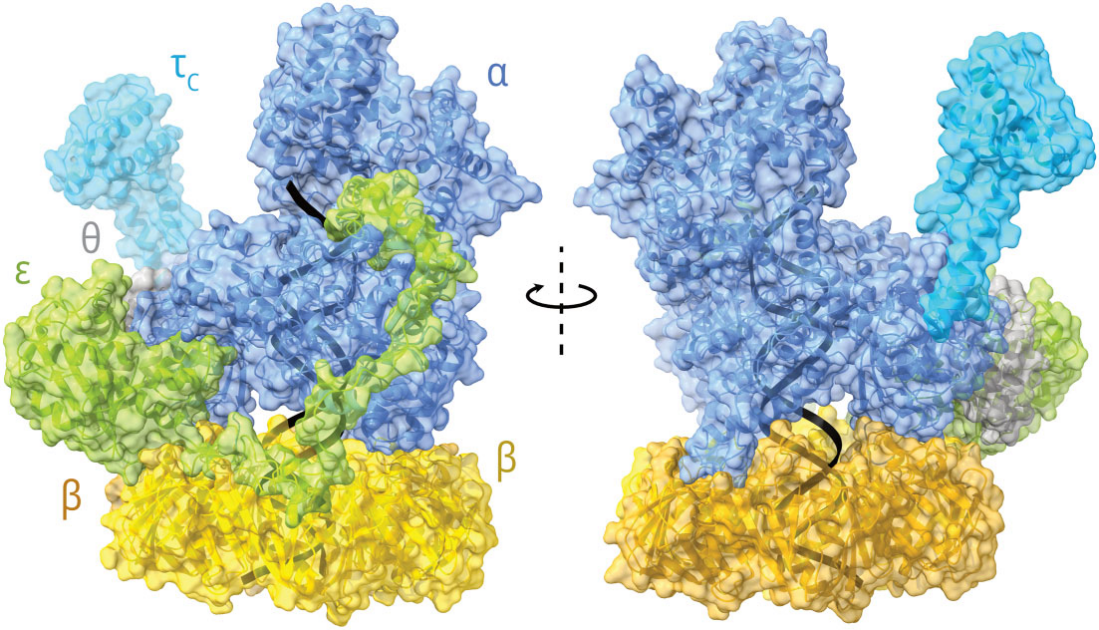
Structure of the Pol III replicative core bound to the β_2_ clamp. The N-terminal parts of the α polymerizing subunit and the ε proofreading subunit occupy the two canonical protein-binding sites in the dimeric β clamp. The C-terminal part of the ε subunit is located close to the α subunit PHP domain, and the two fragments of ε are connected via a flexible glutamine-rich linker. The θ subunit of the core is nested in-between ε and α. The C-terminal fragment of the CLC τ subunit is also shown. The PDB structures 5FKV and 5M1S were used. Modeller was used to model the possible position of the missing ε internal linker.

The clamp has a clear pseudo 6-fold symmetry, with the outer circle composed of β-sheets and the ∼30–35 Å inner circle composed of α-helices. The helices contain many positively charged residues that form electrostatic interactions with DNA that facilitate sliding (Georgescu et al. 2008). Both subunits have canonical protein-binding sites in the form of hydrophobic pockets composed partially of DnaN C-terminal residues. These pockets bind a variety of DNA-interacting proteins possessing specific clamp-binding motifs (CBMs). As previously mentioned, during clamp loading, one protein-binding pocket interacts with the δ subunit of the CLC. During processive replication, both are normally occupied by the replicative polymerase and the exonuclease, the α and ε subunits, respectively (Figs 1A and 2) (Jergic et al. 2013, Toste Rêgo et al. 2013, Fernandez-Leiro et al. 2015), although biochemical evidence suggests that a single binding pocket is sufficient to support clamp loading and DNA synthesis *in vitro* (Scouten Ponticelli et al. 2009). The sliding clamp interacts with many other partners, namely, all DNA polymerases, mismatch repair (MMR) proteins MutS and MutL, and DNA ligase (López de Saro and O’Donnell 2001, Sutton et al. 2001, López De Saro et al. 2006, Maul et al. 2007, Pluciennik et al. 2009, Sikand et al. 2021).

The network of interactions of the β_2_ clamp with its partners is more complicated than singular DNA-binding and protein-binding sites. For example, the protein-binding pocket also interacts with the single-stranded portion of the primed template, with a possible role during clamp loading (Georgescu et al. 2008). Moreover, some clamp-binding proteins, such as DNA polymerase IV, have additional points of contact outside of the hydrophobic cleft (Bunting et al. 2003, Maul et al. 2007, Heltzel et al. 2009, Wagner et al. 2009, Kath et al. 2014). A possibility has been raised that these alternative binding sites might facilitate the rapid exchange of DNA polymerases during replication. Interestingly, β_2_ mutants carrying mutations within the DNA-binding region have been isolated and shown to somehow affect interactions with Pol III or Pol II and Pol IV (Heltzel et al. 2009, Homiski et al. 2021, Berger and Cisneros 2023) and even impair the ability of *E. coli* to tolerate DNA damage (Nanfara et al. 2016).

Apart from increasing the processivity of DNA synthesis, the sliding clamp can also modulate other activities of DNA polymerases, as binding of the β_2_ clamp inhibits Pol I strand-displacement (SD) activity and promotes 5′→3′ exonucleolysis *in vitro*, possibly to avoid excessive DNA resynthesis during Okazaki fragment maturation (Bhardwaj et al. 2018). As the β_2_ clamp also interacts with the ligase, sliding clamps left behind the replication fork might be used by repair enzymes (López de Saro and O’Donnell 2001, Moolman et al. 2014).

Given that around 2000 Okazaki fragments are synthesized in each replication cycle, the demand for the β_2_ clamps far exceeds their cellular levels (Burgers et al. 1981). As the closed clamp conformation is rather stable (Binder et al. 2014), the leftover clamps need to be actively unloaded from the dsDNA. Current evidence points in the direction of the δ subunit of the CLC being the unloader, as addition of δ to an *in vitro* reaction decreases the sliding clamp half-life on DNA from ∼2 hours to around 2 minutes (Yao et al. 1996, Leu et al. 2000). These early results are supported by a more recent single-molecule fluorescence microscopy study where it has been shown that shortly after initiation the number of the DNA-bound β_2_ clamps increases to eventually reach a constant level (∼46, which is ∼50% of the cellular level), maintained until termination, and the half-life of the DNA-bound β_2_ clamp was over 3 minutes (Moolman et al. 2014).

#### The replicative core

Unlike eukaryotes, archaea, and many other bacteria, *E. coli* Pol III’s polymerizing and exonucleolytic proofreading activities are provided by two separate subunits of the replicative core (α and ε, respectively, encoded by *dnaE* and *dnaQ* genes). The third subunit in the core, θ (encoded by the *holE* gene), plays a stabilizing role (Fig. 2) (Taft-Benz and Schaaper 2004). The last decade has significantly expanded our knowledge regarding the structure and interactions within Pol III HE. As previously mentioned, during DNA synthesis, α and ε subunits are both bound to the hydrophobic pockets of the β_2_ clamp. Much insight into the structural arrangement of the α–ε–β_2_ trio came from Cryo-EM studies (Fernandez-Leiro et al. 2015).

**Figure 3.**
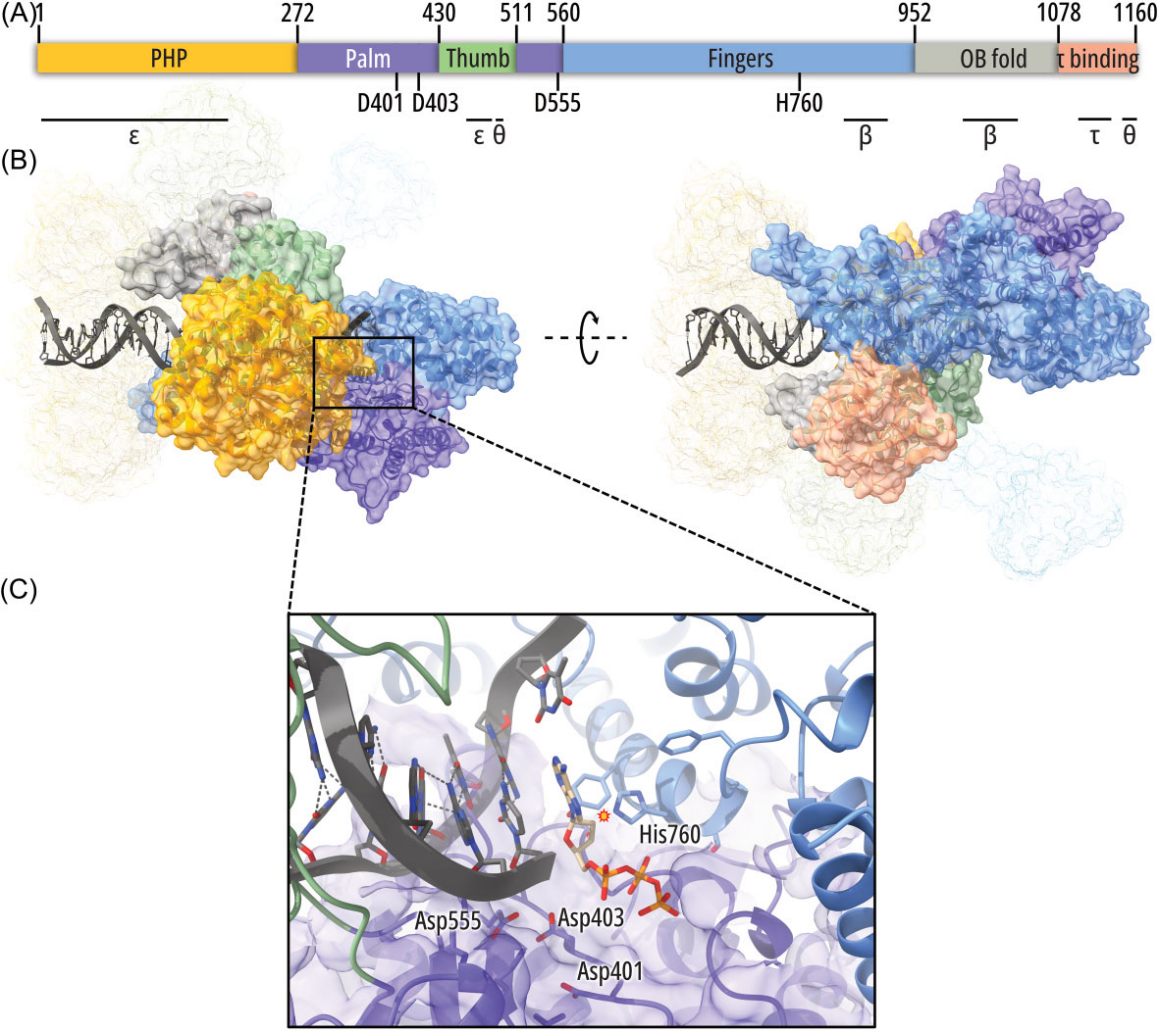
Structure of the α polymerizing subunit of *E. coli* Pol III. Compared to Fig. 2, the structures have been rotated clockwise by 90°. The primary (A) and the ternary (B) structures are shown along with the close-up view of the active site (C); the positions of DNA and other core subunits are visible (see Fig. 3). The three aspartic acids essential for catalysis, as well as the steric gate residue (His760) located in the vicinity of the 2′ carbon of the sugar moiety (star sign), are shown. Major intermolecular contact sites are also marked in (A). The PDB structure 5FKV was used. In (C), the nascent DNA duplex and the incoming nucleotide (dTTP) were modeled based on the PDB structure 3E0D of *Taq* Pol III.

The Pol III α subunit (Pol IIIα) has several domains (Fig. 3A). At the N terminus, there is the polymerase and histidinol phosphatase (PHP) domain, which in some bacteria (that lack the ε subunit) provides the exonuclease activity, although, in *E. coli*, it has been inactivated during evolution. For this reason, it was believed that PHP mostly plays a structural role, although bioinformatic analysis based on sequence alignments suggested that PHP might be a putative pyrophosphatase (Lamers et al. 2006, Barros et al. 2013). This has been confirmed in subsequent studies, where it was shown that pyrophosphate hydrolysis regulates DNA synthesis rate *in vitro* and is important for viability and genome stability (Lapenta et al. 2016). In the central part of Pol IIIα are located the palm, the thumb, and the fingers domains (Fig. 3A). Palm and fingers together participate in creating the active site. The active site fold of Pol IIIα, which is a C-family DNA polymerase, is unlike that of eukaryotic B-family replicases but more akin to that of X-family polymerases such as human Pols β and λ (Parasuram et al. 2018). Several amino acids within the active site are essential for nucleotide selection and catalysis, including the catalytic aspartic acids at positions 401, 403, and 555 in the palm domain and histidine at position 760 in the fingers domain, but also others, including some more distant, residues responsible for correct positioning of amino acids, electrostatic interactions, and proper closing of the active site (Fig. 3A–C) (Parasuram et al. 2018). The nascent DNA duplex is gripped between the thumb domain (which in the primary structure is nested within the palm domain) and the fingers domain (Lamers et al. 2006, Fernandez-Leiro et al. 2015). The fingers domain is longer than in other polymerases but binds DNA loosely, allowing for an unprecedented speed of DNA elongation of ∼700–1000 nt s^–1^ when bound to the β_2_ processivity factor (for comparison, the rate of eukaryotic replication fork progression is ∼25–30 nt s^–1^) (Fig. 3B) (Conti et al. 2007, Sekedat et al. 2010). At the C terminus of the fingers domain, there is the β-binding site, and next to it, there is the oligonucleotide binding (OB) domain, and then the τ-binding region and the very C terminus of Pol IIIα (Fernandez-Leiro et al. 2015).

The Pol III ε subunit (Pol IIIε) can be divided into the big N-terminal catalytic domain (εNTD) and the small C-terminal segment (εCTS). The catalytic domain contains three conserved Exo motifs (I, II, and IIIe). These motifs contain essential residues D12, E14, D103, and D167 that form a DEDD motif, common for many nucleases (DeRose et al. 2002). A fragment between Exo I and Exo II interacts with the θ subunit, while Exo III interacts with the thumb domain of Pol IIIα. At the end of the εNTD, there is the CBM, and the εCTS contains a PHP-interacting fragment connected to the εNTD via a flexible glutamate-rich linker (Fernandez-Leiro et al. 2015). Therefore, Pol IIIε makes two points of contact with Pol IIIα, which is important for DNA transactions during replication as it allows for partial dissociation of Pol IIIε (discussed later, Fig. 2).

The plethora of interactions makes the core a tightly bound complex and increases its affinity to the β_2_ clamp, especially when also bound to DNA. The Cryo-EM structure revealed that upon DNA binding, the core undergoes a significant conformational change with a major shift of the oligonucleotide (OB) domain that forms another point of contact with the sliding clamp between the polymerase and the exonuclease (Fernandez-Leiro et al. 2015). Accordingly, the core–β_2_–DNA complex is much more stable than the core with the clamp alone (Naktinis et al. 1996). But the core subunits also stimulate each other as the core is *in vitro* more proficient in both synthesis and exonucleolysis than α and ε alone (described in more detail in Lewis et al. 2016).

#### The ssDNA-binding protein

The SSB protein coats single-stranded fragments of DNA produced by the helicase, thereby protecting them from degradation and preventing formation of DNA secondary structures that could interfere with replication (Fig. 1A). SSB is a homomeric complex composed of four subunits encoded by the *ssb* gene. The N-terminal OB domain of the SSB subunit participates in DNA binding, whereas the C-terminal part contains a long, disordered interdomain linker (IDL) that is important for cooperativity, and the conserved 9-amino-acid-long tail (SSB-Ct) that enables interactions with many different partners (in binding many of which IDL also plays a role; reviewed in Oakley 2019, Bianco 2021).

Depending on salt concentration and relative SSB and DNA concentrations, three SSB binding modes to DNA have been observed that differ in the number of nucleotides per tetramer: SSB_35_, SSB_56_, and SSB_65_ (Lohman et al. 1986, Bujalowski and Lohman 1989, Ferrari et al. 1994). SSB_35_ displays high nearest-neighbor binding cooperativity mediated by the interaction between the IDL and the OB domain of the neighboring SSB_35_, and the SSB–DNA complexes form large clusters observed in electron microscopy (Griffith et al. 1984, Kozlov et al. 2015). On average, two OB sites are involved in complex formation. A recent study reveals the presence of a conserved surface close to the DNA-binding site that interacts with a DNA fragment that “bridges” two tetramers. This interface is important for linking SSB tetramers and thus forming higher-order complexes in SSB_35_ binding mode (Dubiel et al. 2019). In contrast, the crystal structure of SSB65 shows DNA wrapped around four SSB subunits and thus occupying all available OB sites in a manner described as a “basketball seam” (Raghunathan et al. 2000). These complexes seem to form octamers visualized as beads on DNA in electron microscopy and are characterized by low binding cooperativity.

Recent investigations utilizing single-molecule force and fluorescence spectroscopy and optical tweezer methods revealed the mechanisms of DNA wrapping and unwrapping. Binding seems to occur through intermediate states where initially eight and then 17 nt are wrapped, likely mediated by an initial DNA interaction with the W54–R56 cluster (Suksombat et al. 2015, Naufer et al. 2021). Wrapping is linear and goes through SSB_8_, SSB_17_, SSB_35_, and SSB_56_ to finally reach the SSB_65_ stage, while unwrapping occurs in the opposite direction (Suksombat et al. 2015). Notably, single-molecule studies show that SSB is dynamic on ssDNA and able to not only switch binding modes but also change its position (translocate) (Roy et al. 2007, 2009, Zhou et al. 2011). Translocation, which is a fast process, occurs via a mechanism called reptation, a snake-like movement that is typical of long polymers: SSB remains mostly bound to DNA, while short 2–5-nt fragments are unwrapped and quickly replaced by another fragment of ssDNA. In the process, a small bulge of unwrapped DNA travels around the SSB tetramer, effectively leading to SSB translocation with respect to DNA (see the Supplementary video in Zhou et al. 2011). Other observations suggest that SSBs are initially deposited on ssDNA (e.g. produced by the helicase) and swiftly wrapped around 65-mers subject to ssDNA availability, but when SSB starts to build up, the binding mode changes to SSB_35_, which is a much slower process and probably mediated by high binding cooperativity (Naufer et al. 2021). Based on these observations, a model of SSB dynamics during DNA replication has been proposed. When free ssDNA shortens, e.g. due to ongoing DNA synthesis or RecA filamentation, SSBs are first pushed together (due to rapid translocation), overcrowded, and finally ejected from DNA, after which they might quickly reassociate with newly produced ssDNA (Naufer et al. 2021). The oversaturation might stimulate the rates of unwrapping and dissociation, which are normally quite low. SSB translocation might be assisted by the movement of the replication machinery, although an alternative explanation is that the replicase actively dislocates the SSB (Cerrón et al. 2019).

SSB’s interactome is extensive and comprises primase; DNA Pols II, III, IV, and V; nucleases such as ExoI, Exo IX, and RNase HI; helicases such as DinG, RecG, or RecQ; primosome proteins PriABC; topoisomerase III; and other DNA repair proteins such as AlkB, RadD, RecO, RecJ, and Ung (Arad et al. 2008; reviewed in Bianco 2021). Most of these proteins have an OB-fold that mediates SSB interactions. Thus, SSB can be viewed as an important hub that orchestrates replisome transactions and facilitates DNA maintenance (Shereda et al. 2008). This perspective has been expanded using more recent discoveries in another section of this manuscript.

#### The auxiliary DNA polymerases

DNA polymerase I (Pol I) encoded by the *polA* gene is not an integral part of the replisome but actively participates in DNA replication. The C-terminal segment of Pol I (Klenow fragment) contains the polymerase and the 3′→5′ exonuclease, while the N-terminal segment connected via a flexible linker provides the 5′→3′ exonuclease and the endonuclease activities (see, e.g. Lewis et al. 2016 for more details). Pol I synthesizes patches of DNA to replace RNA primers in a process called Okazaki fragment maturation (Fig. 1A). This allows for the subsequent ligation of Okazaki fragments by the DNA ligase. Pol I is also a versatile repair polymerase participating in short-patch repair pathways such as nucleotide excision repair (NER), base excision repair, very short patch repair, and ribonucleotide excision repair (RER) (McDonald et al. 2012, Robertson and Matson 2012). Indeed, Pol I is well-equipped to accomplish these tasks as it is proficient in 3′→5′ exonucleolytic, 5′→3′ exonucleolytic, endonucleolytic, and SD activities and can perform nick-translation, gap-filling, and SD synthesis. On the other hand, the processivity of Pol I is low (15–20 nt synthesized per binding event; Bambara et al. 1978) unless bound to the β_2_ clamp (López de Saro and O’Donnell 2001), which in turn inhibits its SD activity and promotes nick-translation and early ligation *in vitro* (Bhardwaj et al. 2018). This might explain why Pol I is not believed to be the major contributor to the MutSLH-dependent MMR pathway.

An insight into the mechanism of Pol I-mediated Okazaki maturation was provided in recent studies that utilize single-molecule microscopy, *in vitro* biochemistry, and structural biology approaches (Craggs et al. 2019, Pauszek et al. 2021, Botto et al. 2023). The first step is the handover of the primer terminus from Pol III to Pol I. How Pol I is recruited to the substrate and whether Pol I binds to a nick or a gap is not clear. *In vitro* experiments suggest that it might be a nick based on processive Pol III-mediated replication (Botto et al. 2023), but the alternative model implies that Pol III might abandon the Okazaki fragment before it is replicated up to the next primer, thus leaving a gap for Pol I to fill; *in vivo* experiments are consistent with this idea (Graham et al. 2017). Another question is whether Pol I utilizes the β_2_ clamp during Okazaki fragment maturation. This seems likely as the clamp was shown to prevent excessive Pol I SD activity *in vitro* (Bhardwaj et al. 2018), and live-cell imaging revealed that dozens of clamps are bound to DNA in actively dividing cells, each for over 3 minutes (Moolman et al. 2014). The steady-state number of clamps is reached in less than 10 minutes and remains so for over 60 minutes. These clamps are likely left behind by the lagging-strand (and less frequently the leading-strand) polymerase subassembly at a perfect place to be utilized by Pol I (and also other repair proteins). However, the β_2_ clamp does not seem to stimulate Okazaki fragment maturation *in vitro* (Botto et al. 2023).

A structure of Pol I in complex with the template, upstream, and downstream strands shows the template is bent by ∼120° at the Pol I fingers domain, which leads to partial (1–2 nt) fraying of the RNA substrate, with the unpaired bases interacting with Arg781 and Phe771. This substrate is displaced by the fingers during the Pol I translocation (Craggs et al. 2019, Botto et al. 2023). As Pol I replicates up to the end of the primer, it simultaneously cleaves the flap. It has been shown using FRET microscopy that the flap can spontaneously transfer between the polymerase and the endonuclease (Pauszek et al. 2021), which likely facilitates primer nucleolysis. However, an *in vitro* assay that utilized an RNA-primed DNA substrate showed that Pol I is a very proficient junction nuclease, cleaving on the 3′ side of the last ribonucleotide with high specificity (Botto et al. 2023). How this specificity is achieved is not clear, but it stands to reason that Pol I recognizes some kind of additional signal that triggers endonucleolysis. Interestingly, as the endonuclease reaction in (Botto et al. 2023) was carried out in the absence of dNTPs, meaning that the polymerase could not translocate, it seems likely that during Okazaki fragment maturation, the cut is introduced before Pol I reaches the end of the flap. Thus, a plausible scenario is that Pol I nicks the RNA–DNA junction at the beginning or during extension of the upstream DNA strand, which is accompanied by flap cleavage, and then terminates at the nick, which is subsequently ligated by the LigA ligase (Botto et al. 2023).

**Figure 4.**
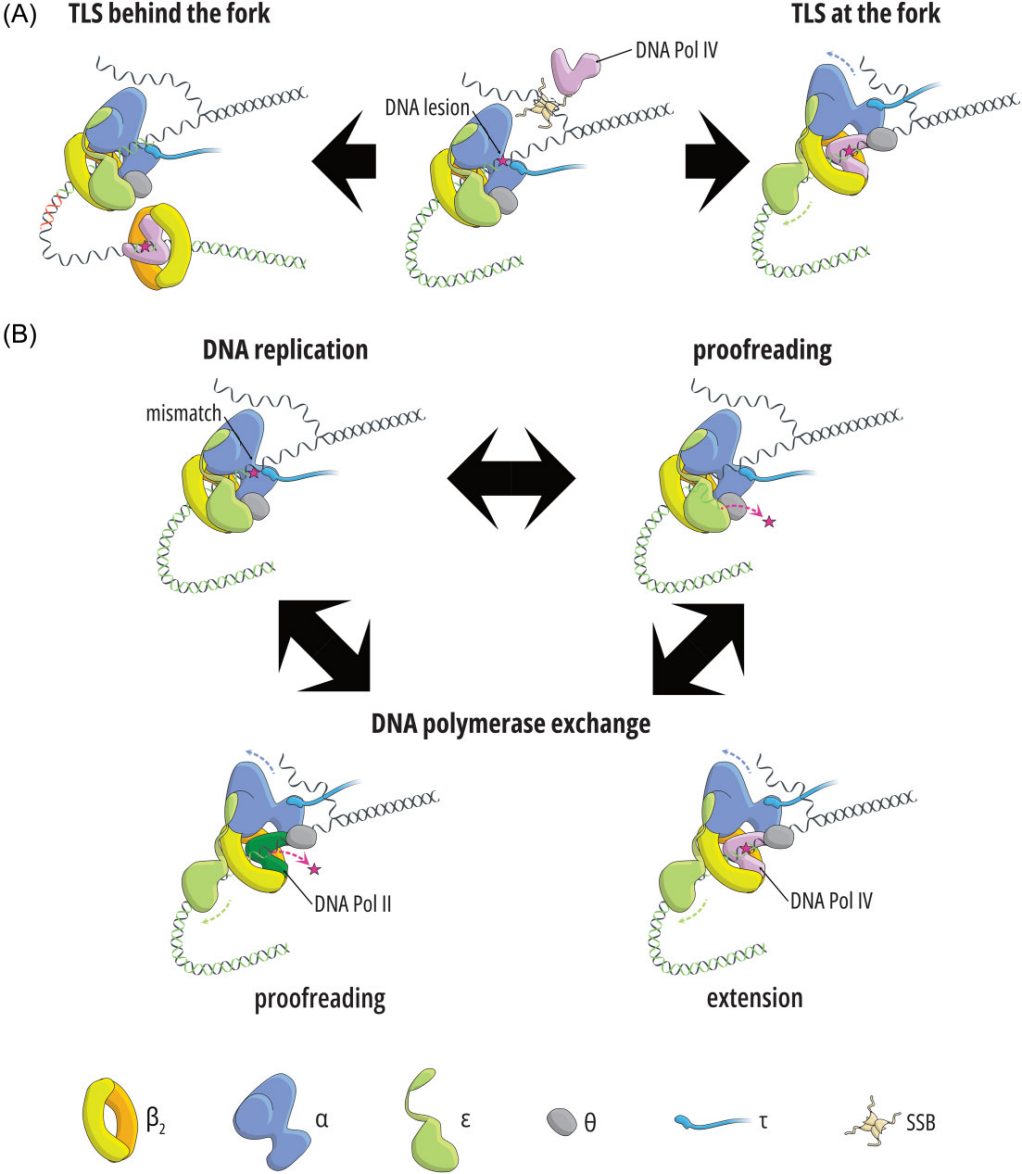
The fate of lesions and mismatches at the replication fork. (A) When the replicase encounters a DNA lesion, specialized TLS DNA polymerases are recruited to help replicate past the damage. This might happen either at the replication fork via polymerase switching, or behind the replication fork after polymerase dissociation and subsequent repriming downstream the lesion. (B) Mismatches introduced by the replicase might be removed by the intrinsic proofreading activity provided by Pol IIIε. Alternatively, partial dissociation of Pol III allows for recruitment of auxiliary DNA polymerases, with the outcome (excision or extension of the mismatch) depending on the associated polymerase (high- or low-fidelity).

Pols II, IV, and V (encoded by *polB, dinB*, and *umuDC*, respectively) are the three *E. coli* DNA polymerases that are involved in TLS (Fig. 4A) (see Maslowska et al. 2019, Fujii and Fuchs 2020 for review). Pol II is a B-family polymerase that can bypass abasic sites and acetylaminofluorene adducts (AAF-dG). Y-family Pol IV and Pol V are specialized in dealing with minor groove and major groove lesions, respectively. For example, Pol IV can bypass alkyl adducts, whereas Pol V performs TLS on UV lesions (Tessman and Kennedy 1994, Napolitano et al. 2000, Fujii and Fuchs 2007, Robinson et al. 2015, Wang et al. 2021). Unlike Pol V, Pols II and IV are normally present in the cell at detectable concentrations. These levels are five times higher for Pol IV (∼50 versus ∼250 molecules/cell), but Pol II has a higher affinity for the β_2_ clamp (Bonner et al. 1992, Wagner et al. 2000, Sutton 2010). Given that Pol II is a high-fidelity, exonuclease-proficient DNA polymerase, while Pols IV and V are not, it is possible that one of the cellular roles of Pol II is to limit Pol IV’s mutator potential. Additionally, genetic evidence indicates that Pol II may serve as a backup replicase when Pol III has trouble continuing the replication (Banach-Orlowska et al. 2005, Fijalkowska et al. 2012).

Upon DNA damage, the cellular levels of Pols II, IV, and V can be further elevated due to the activation of the so-called “SOS” response, which results in the upregulation of specific genes (Fernández de Henestrosa et al. 2000, Courcelle et al. 2001). Other consequences of SOS induction are, for example, activation of NER and HR (reviewed in Bell and Kowalczykowski 2016). The slowdown of fork progression upon DNA damage allows for the binding of RecA protein to DNA. RecA has several cellular functions: it is a protein involved in HR, but it is also a mediator of the SOS response and SOS-dependent mutagenesis. RecA must compete for ssDNA with SSB, which normally coats exposed DNA regions. Because of SSB’s high affinity to ssDNA, binding of RecA to DNA is a timely process unless aided by other complexes such as RecFOR (Morimatsu and Kowalczykowski 2003). RecFOR can target DNA repair to ssDNA gaps, and recent studies suggest that this activity might be mediated by RecF interaction with the β_2_ clamp, particularly with ones associated at abandoned replication intermediates when the replicase stalls, dissociates, and continues replication downstream damage (Henry et al. 2023). In line with these findings, RecF frequently colocalizes with the replisome (Henrikus et al. 2019). Other important factors in SSB displacement are the diffusion of SSB, which leads to exposure of uncoated fragments (Roy et al. 2009), and SSB–RecA interaction, which modulates filament formation (Wu et al. 2017). Creation of the nucleoprotein filament (often denoted as RecA*) promotes proteolysis of LexA, which is a repressor of the SOS system that governs the transcription of specific genes such as the *umuDC* operon encoding Pol V (Shibata et al. 1981, Shinagawa et al. 1988, Patel et al. 2010, Cory et al. 2024). *lexA* and *recA* genes are LexA–regulated and RecA–deregulated themselves such that SOS induction is suppressed quickly when replication fork progression is restored, and RecA* filaments are no longer forming.

Pol V is a heterotrimeric protein composed of the UmuC catalytic subunit and two noncatalytic UmuD′ subunits. Pol V exhibits a strong mutator potential and is subject to an astonishingly complex system of control comprising transcriptional, temporal, spatial, and biochemical elements of the regulation (reviewed in Goodman et al. 2016, Jaszczur et al. 2016). Firstly, Pol V is normally undetectable in the cell. After LexA– and RecA–dependent transcription starts, which happens ∼15 minutes after SOS activation, UmuC and UmuD_2_ proteins are quickly degraded by the Lon protease, delaying protein accumulation to ∼45 minutes after SOS activation when the rate of translation overcomes the rate of proteolysis (Gonzalez et al. 1998). Secondly, as revealed by the more recent single-cell microscopy studies, when UmuC and UmuD_2_ accumulate, UmuC is sequestered at the cell membrane awaiting UmuD_2_ autoproteolytic activation (the activated dimer is denoted UmuD′_2_), allowing for the assembly of Pol V (UmuD′_2_C) ∼1 hour after SOS induction (Robinson et al. 2015). However, activation of Pol V additionally requires the binding of RecA and ATP, and this active complex is called the Pol V mutasome (Pol V mut). Thirdly, Pol V mut is the slowest DNA polymerase (∼0.29 nt s^–1^), and its processivity strongly depends on SSB (∼25 nt per binding event when bound to the β_2_ clamp; ∼200 nt per binding event when SSB is also present) (Tang et al. 2000, Pham et al. 2001, Karata et al. 2012). Fourthly, as the SOS signal subsides, UmuD binds to UmuD′, forming a heterodimer, and then UmuD′ is degraded by ClpXP, while UmuD and UmuC are degraded by Lon, as already mentioned (Frank et al. 1996, Gonzalez et al. 2000). These elements ensure that Pol V expression and action, and therefore its mutator effect, are kept to a minimum. Interestingly, in the *recA730* genetic background, where RecA_E38K exists in a constitutively induced state due to the more efficient competition with SSB, most of the regulation is circumvented such that Pol V is constantly expressed without any DNA damage, promoting high levels of spontaneous mutagenesis, especially when combined with a LexA deficiency (e.g. *lexA51*) (Watanabe-Akanuma et al. 1997, Niccum et al. 2020).

#### Other activities near the replisome

Replisomes frequently encounter different insults that block or slow down their progression and have the potential to affect replication fidelity and genome stability. DNA lesions can be dealt with on the fly using TLS polymerases or by activating the SOS system, which is mediated by the RecA protein. These have been described in the previous section. However, an equally important source of replication obstacles is the never-ending DNA metabolism and maintenance. Replisomes may clash with proteins associated in front of the fork or undissociated transcripts, forming structures known as R-loops. The primary enzymes responsible for the removal of protein or RNA adducts from DNA are Rep helicase and RNase HI, respectively. Importantly, both might be viewed as transient, auxiliary components of the replication machinery, as interactions with replisome subunits enrich them at the sites of ongoing replication.

The monomeric Rep helicase translocates 3′→5′ on the leading-strand template, in contrast to the replicative helicase DnaB (Korolev et al. 1997). Rep is proficient at displacing DNA-bound proteins such as the RNA polymerase (RNAP) but does not unwind dsDNA (Brüning et al. 2018, Hawkins et al. 2019). Another *E. coli* helicase with partially redundant activity is UvrD, a component of MMR and the NER machinery, as loss of both is lethal under fast growth conditions. However, only Rep was shown to physically interact with the replisome (Atkinson et al. 2011), and unlike in the case of other auxiliary helicases, loss of Rep significantly affects cell growth, for which this interaction is crucial (Atkinson et al. 2011). In a recent live cell imaging study, it has been proposed that Rep monomers might occupy all six DnaB subunits, and the interaction is stochastic and dynamic (Syeda et al. 2019). Other studies suggest a lower occupancy (Whinn et al. 2023). This implies that DnaB may serve as a launching pad for Rep probes, where Rep constantly surveils DNA for protein roadblocks and performs quick displacement (Fig. 1A) (Syeda et al. 2019, Whinn et al. 2023). We note in passing that UvrD also colocalizes with the replisome to aid protein displacement, but no specific recruitment factors in this context have been identified (Wollman et al. 2024).

RNase HI, which will be discussed in detail in the section dedicated to ribonucleotide repair in DNA, is an endoribonuclease that cleaves RNA transcripts in R-loops. It has been shown that RNase HI interacts with the C-terminus of SSB, which is important for the stimulation of its activity (Petzold et al. 2015). However, more recent studies revealed that this interaction is responsible for RNase HI colocalization with the replisome (Wolak et al. 2020). A mutant strain in which this interaction is eliminated is characterized by slowed growth and activation of the DNA damage response when combined with a Rep deficiency, and this phenotype is dependent on the level of ongoing transcription, indicating that RNase HI enrichment near the replication fork is important for R-loop removal in front of replication fork (Fig. 1A) (Wolak et al. 2020). Importantly, there are other enzymes capable of R-loop repair, including DinG helicase which was shown to unwind R-loops (Voloshin and Camerini-Otero 2007). DinG is also stimulated by the interaction with SSB (Cheng et al. 2012), but whether this protein is deposited at the replisome similar to RNase HI is currently unknown.

Replisome activity leads to the accumulation of topological stress that is relieved by type II topoisomerases acting both in front of and behind the replication fork (Bush et al. 2015). Positive supercoiling due to DNA unwinding by the helicase in front of the fork is relaxed by gyrase. Topoisomerase IV (topo IV) may also play a role in this process, but it is essential behind the fork for disentangling daughter chromosomes that become catenated due to migration of positive supercoils from the front (Sissi and Palumbo 2010, Ashley et al. 2017).

Topo IV, a tetramer composed of ParC_2_ (responsible for DNA binding and catalysis) and ParE_2_ (ATPase), can work on a variety of substrates, including positive and negative supercoils as well as catenates (Bush et al. 2015). Topo IV has been shown using live imaging to colocalize with the structural maintenance of the chromosome (SMC) complex MukBEF that is indispensable for proper positioning and segregation of sister chromosomes (Nicolas et al. 2014, Zawadzki et al. 2015). Topo IV–MukBEF interaction is probably important for decatenation near *oriC*s in preparation for their subsequent separation. However, Topo IV also interacts with the SeqA protein that trails behind the fork where it binds hemimethylated GATC sequences (Fig. 1A) (Kang et al. 2003). SeqA plays multiple roles: it limits overinitiation of DNA replication (Pedersen et al. 2017), prevents premature methylation of DNA by the Dam methylase, enabling the activity of MMR (Fig. 1C) (Kang et al. 1999), promotes early cohesion of sister chromosomes, which is important for proper segregation (Joshi et al. 2013), and orchestrates Topo IV action along the replicated DNA (Helgesen et al. 2021). The exact mechanism is not clear, but it is possible that Topo IV binds SeqA clusters on the replisome-distal side, where it catalyzes chromosome disentanglement.

Gyrase is composed of a single GyrA (DNA binding) and two GyrB (ATPase) subunits. Gyrase is not efficient at decatenation, and is thus believed to be primarily responsible for the introduction of negative supercoils in front of the fork, at which it is more efficient than Topo IV (Fig. 1B) (Bush et al. 2015, Ashley et al. 2017). A single-molecule study of gyrase distribution in the cell suggests that besides the many gyrase molecules bound across the chromosome likely to maintain steady-state levels of supercoiling, there is also an enrichment near the replication fork with increased dwell time, suggesting processive action in front of the fork (Stracy et al. 2019). Intriguingly, the combined rate of relaxation by gyrase and Topo IV, as observed in these studies, is not sufficient to keep up with the rate of DNA replication (Stracy et al. 2019), and additional regulatory elements called Replication Risk Sequences have been identified recently. During replication, these GC-rich sequences promote formation of single-stranded gaps on the lagging strand to control supercoil formation (Pham et al. 2024). Moreover, no specific factors that would recruit gyrase to the replisomes have been identified, nor is it known whether such factors exist; it is possible that the already bound gyrase units are engaged for processive relaxation of topological stress in front of the fork.

### The dynamics of the replisome

Multiple lines of evidence suggest that the *E. coli* replication fork is a dynamic entity that undergoes many transactions involving most of its components. This includes the dynamic nature of the SSB protein on DNA, the cycles of primer synthesis and clamp loading, and also the exchange of different DNA polymerases at the replication fork and of the replicase holoenzymes themselves. These will be discussed in the following paragraphs.

#### The helicase–primase–replicase axis

DnaB helicase is the central protein of the replisome that links the activities of the DnaG primase and the Pol III holoenzyme. However, the timing of DNA unwinding, priming, and synthesis need to be tightly coordinated to avoid uncoupling and replication failure. An important layer of regulation of DnaB_6_ relies on its cycling between the two conformational states, dilated and constricted, that vary in properties. For instance, in the dilated conformation, the rate of unwinding is lower than in the constricted form (Strycharska et al. 2013). Additionally, priming activity is stimulated by DnaB_6_ in the dilated form when unwinding is also slower; constricted DnaB_6_ does not support priming (Strycharska et al. 2013, Monachino et al. 2020). Likewise, the Pol III τ subunit interacts strongly with the dilated DnaB_6_, significantly increasing the rate of unwinding (Monachino et al. 2020).

Another layer is primase binding itself. During translocation, DnaG binding sites are constantly disrupted are reformed, suggesting that the primase does not bind the helicase for the whole period or primer synthesis (Manosas et al. 2009, Itsathitphaisarn et al. 2012), consistent with the fact that during catalysis, both move in opposite directions. Notably, while analysis of the strength of the DnaB–τ interaction in solution suggests that the free helicase exists predominantly in a state between constricted and dilated, binding of DnaG markedly increases the strength of this interaction, indicating that DnaG binding to the helicase induces a switch to the dilated conformation, which promotes its interaction with the holoenzyme (Monachino et al. 2020). As DnaG is likely to be forcibly ejected from the helicase during translocation (Manosas et al. 2009, Itsathitphaisarn et al. 2012), and the rate of unwinding is lower when there is no CLC (Strycharska et al. 2013), it is possible that the pace at which DnaB_6_ produces ssDNA is dynamically adjusted depending on the presence and/or strength of interaction with τ. Indeed, the helicase slows down by 80% in response to leading-strand–replicase pausing (Graham et al. 2017), and conformational transactions are a plausible explanation for this phenomenon. However, the binding of DnaG does not lead to helicase pausing (Monachino et al. 2020), and thus, the exact mechanism of how lagging-strand synthesis is coordinated with priming and unwinding remains to be uncovered.

#### Replicase exchange at the replication fork

Pol III HE is very processive, capable of synthesizing thousands of kilobases of DNA per binding event at a very high speed *in vitro* and replicating ∼2.3 Mb (i.e. half of the chromosome per replisome) of DNA in around 40 minutes *in vivo*. Accordingly, the textbook view of DNA replication has been that the replicase remains steadily bound at the replication fork for the period of DNA replication. However, a body of evidence gathered from live cell imaging suggests that the DnaB_6_ helicase is the only stable element of the replisome, remaining bound at the replication fork for ∼30 minutes (Beattie et al. 2017, Spinks et al. 2021). In contrast, Pol III* (i.e. Pol III HE *sans* the β_2_ clamp) at the replication fork frequently exchanges with free subassemblies from the cytosol every several Okazaki fragments both *in vitro* and *in vivo* (Beattie et al. 2017, Lewis et al. 2017). As Pol III goes through cycles when it binds the helicase either strongly or weakly (Monachino et al. 2020), and replisome pausing every few seconds was observed *in vitro* (Graham et al. 2017), it is possible that it is during that conformational switch that Pol III* exchange takes place.

This observation raises several questions. First, is the leading strand replication truly discontinuous? An answer to this problem was provided in a study where replication intermediates from actively dividing cells were separated at high resolution using sucrose gradients (Cronan et al. 2019). These intermediates were approximately ∼80 kb long on the leading strand and ∼1.2 kb long on the lagging strand. Thus, the leading strand is seemingly replicated in a chemically continuous manner, with the possible exception of encounters with different insults that lead to either repriming below the block or replication fork collapse and subsequent reassembly, depending on whether the helicase can accommodate them. However, Pol III* is now known to pause and dissociate from the 3′ terminus, which is then picked up by another replicative complex, and therefore the leading-strand replication is also kinetically discontinuous (Graham et al. 2017).

**Figure 5.**
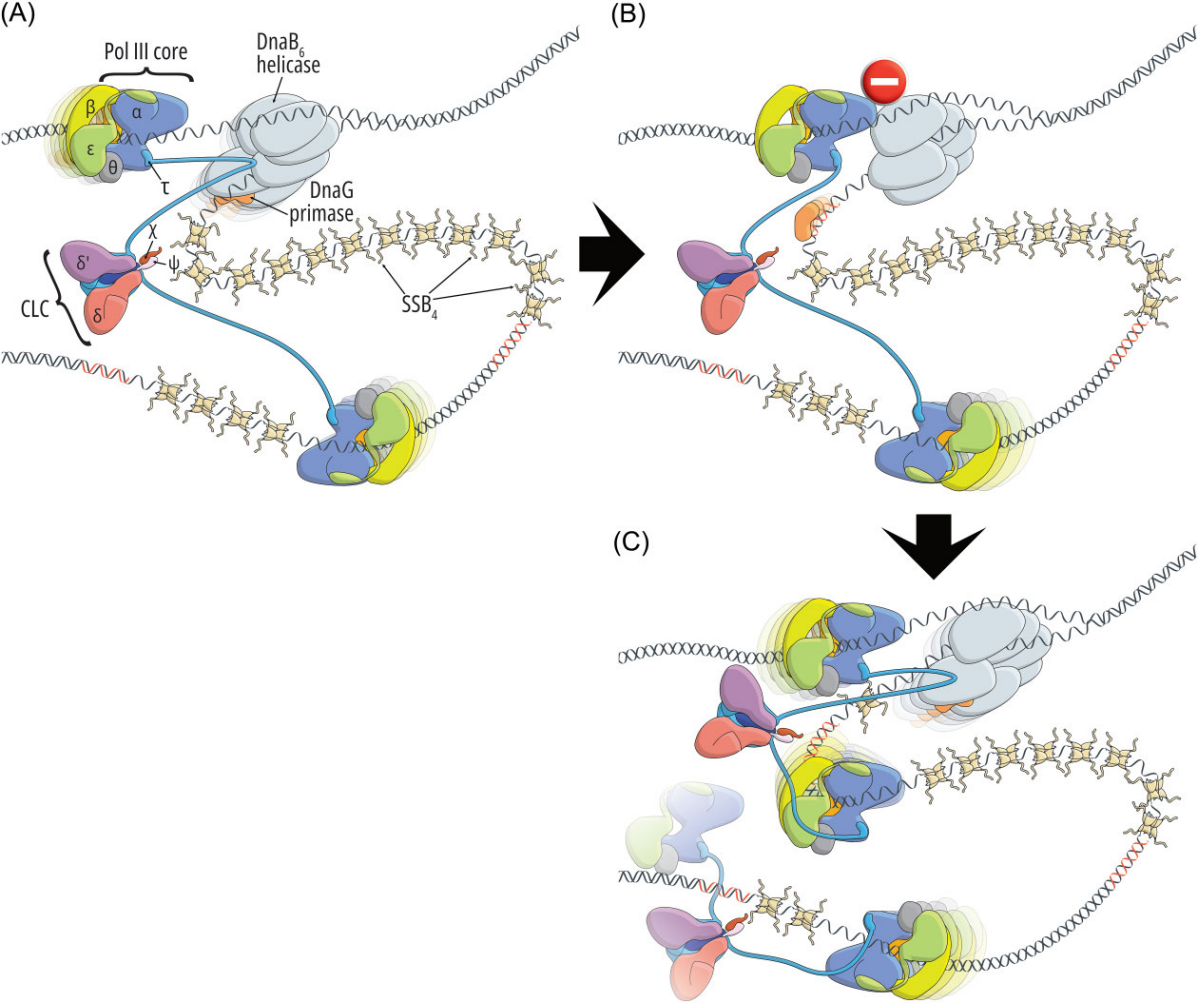
A theoretical model of replisome dynamics during leading- and lagging-strand replication. (A): Pol III* is tightly bound to the helicase, allowing for fast and processive simultaneous unwinding and replication of both DNA strands. (B): When DnaG primase is ready, it disembarks from the helicase and proceeds to synthesize a primer, guided by the interaction with SSB. The loss of DnaB–DnaG interaction induces conformational changes in the helicase that destabilize the interaction with Pol III*. Pol III core subassembly on the leading strand pauses, and the helicase slows down. (C): A new DnaG subunit binds to DnaB_6_, and a new Pol III* is recruited. On the lagging strand, the CLC of the new Pol III* displaces the primase, loads the β_2_ clamp, and the synthesis of the next Okazaki fragment begins. On the leading strand, the second core binds to the β_2_ clamp left behind by the previous Pol III* and resumes replication. If necessary, the previous Pol III* may quickly finish the replication of the previous Okazaki fragment.

Another interesting question is to what extent leading- and lagging-strand replication are coordinated. A certain level of coordination seems necessary as replication forks need to converge timely, and all gaps need to be filled as under conditions of fast growth, these nascent DNAs are also templates for the next advancing forks. This problem predominantly concerns lagging-strand replication, which requires multiple cycles of dissociation, priming, clamp loading, and reassociation. One might expect that the replisome would be regulated in response to these challenges, and yet, no specific signals have been discovered, and it seems that the lagging strand has no trouble keeping up with the leading strand even when priming frequency is artificially altered, at least *in vitro* (Graham et al. 2017). This led to the proposal that the leading- and lagging-strand replicase subassemblies work independently of each other. In principle, one may hypothesize that the leading-strand pausing and replicase exchange could be the mechanisms that ensure the temporal coordination of both DNA strands. For example, if the lagging-strand core had trouble completing Okazaki fragment synthesis, Pol III* dissociation from the helicase would allow for the replication of the lagging-strand gap to be completed by this Pol III complex, while another copy of the holoenzyme associates to the helicase and resumes replication (Fig. 5). Indeed, according to more recent calculations, the rate at which Pol III* exchanges correlates with the time required for replication of a single Okazaki fragment (see the section “Discussion” in Monachino et al. 2020). It is also possible that the third core in the holoenzyme participates in lagging-strand replication, facilitating a quick switch or even simultaneous replication of two Okazaki fragments (Montón Silva et al. 2015, Beattie et al. 2017, Xu and Dixon 2018). Any of these could contribute to diminishing the supposed bottleneck resulting from the lagging-strand replicase cycling.

It is worth noting that *in vivo* data regarding the ssDNA gap ratio between the leading- and lagging DNA strands are conflicting. In one WGS-based study, in which *E. coli* cells expressed the CTD of the APOBEC3G deaminase that specifically converts dC to dU in a ssDNA substrate, a 2-fold strand bias toward the C in the lagging-strand template was observed upon *ung* deletion (the gene encoding Ung glycosylase that repairs such lesions), suggesting that this strand is more accessible to APOBEC3G (Bhagwat et al. 2016). In another investigation, isolated gDNA was treated with bisulfite, which also deaminates deoxycytidine on ssDNA; here, the sequencing data showed no bias (Pham et al. 2022). More studies are needed to determine whether the lagging-strand machinery has any trouble keeping up with the leading-strand replication.

#### Polymerase switching at the replication fork

Apart from the exchange of identical Pol III* complexes, replisomes may also occasionally switch from Pol III-dependent to accessory-polymerase-dependent DNA replication (Pols II, IV, and V). Key evidence for this phenomenon came from genetic assays showing that that Pol II, IV, and V mutational signatures can be observed *in vivo* when an exonuclease-deficient mutant of Pol II is expressed, Pol IV is overproduced, or Pol V is constitutively activated (Maliszewska-Tkaczyk et al. 2000, Kuban et al. 2004, 2005, Banach-Orlowska et al. 2005, Curti et al. 2009). Moreover, a simultaneous defect in proofreading by Pol III and Pol II has a synergistic effect on mutation rates, indicating that Pol II normally repairs errors introduced by the replicase (Banach-Orlowska et al. 2005). The effect of the accessory polymerases is exacerbated in strains expressing mutant Pol III with an increased propensity to dissociate from the primer terminus (Makiela-Dzbenska et al. 2019). These data corroborate biochemical observations (1) that all accessory DNA polymerases interact with the β_2_ clamp (Sutton 2010, Fijalkowska et al. 2012, Yang and Gao 2018, Fujii and Fuchs 2020), (2) that Pol II can utilize the CLC for DNA synthesis (Bonner et al. 1992, Kath et al. 2015), and (3) that Pols III and IV can be simultaneously bound to the clamp (Indiani et al. 2005).

The unique structure of the Pol III core might facilitate the polymerase exchange. The β_2_ clamp has two canonical binding sites, one on each subunit, typically occupied by the α and ε subunits (Fig. 2) (Jergic et al. 2013). However, the β–ε interaction is relatively weak, and the exonuclease can frequently dissociate from the clamp while still being bound to the α subunit via its C-terminal part (Toste Rêgo et al. 2013, Whatley and Kreuzer 2015). The partial dissociation is possible due to a flexible linker that connects the N and C termini (Fig. 2). Increasing the strength of the β–ε interaction, i.e. making Pol IIIε less susceptible to dissociation resulted in SOS induction and a defect in TLS, suggesting problems with the efficient repair of lesions and replication fork stalling, because the TLS polymerases have trouble to effectively substitute for Pol III at the fork (Whatley and Kreuzer 2015). Conversely, weakening the interaction increases TLS (Chang et al. 2019). Therefore, the ε subunit serves as a gatekeeper that regulates the access of Pols II, IV, and V to the replication fork (Fig. 4A and B) (Jonczyk et al. 1988, Kath et al. 2014, 2015, Thrall et al. 2017, Chang et al. 2019, Tuan et al. 2022). Interestingly, it has been shown that the interaction with SSB might enrich Pol IV near the replication fork, facilitating quick TLS when Pol III stalls (Chang et al. 2022, Thrall et al. 2022). On the other hand, single-cell imaging revealed that Pol V foci do not colocalize strongly with the replisome upon UV irradiation, suggesting that TLS occurs behind the replication fork (Robinson et al. 2015). Similar observations were made with Pol IV when a different type of DNA lesion was induced (Henrikus et al. 2018). Under this scenario, replication progresses after repriming downstream the damage. Hence, repriming with TLS behind the fork and Pol IV-mediated TLS at the fork seem to be two competing mechanisms, with the outcome likely governed by the type of lesion and the strength of the β–ε interaction (Fig. 4A) (Marians 2018, Chang et al. 2019, Sale 2022), and perhaps also simple stochastic competition. Importantly, when the SOS system is constitutively activated, Pol V foci colocalize with the replisome (Robinson et al. 2015), but it is unknown whether polymerase switching, in this case, is also mediated by Pol IIIε dissociation or some other mechanism, especially given that Pol V activation requires mutasome assembly. An alternative model of polymerase exchange that involves a complete dissociation of the Pol III core from the β_2_ clamp and the subsequent association of an auxiliary polymerase has been proposed (Zhao et al. 2017).

#### The role of SSB in organizing replisome transactions

ChIP-seq analysis reveals that SSB is ubiquitous on the lagging DNA strand (Pham et al. 2023). Such an abundance has several consequences. First, as argued in the previous sections, SSB needs to be frequently rearranged and/or displaced during replication. For example, it is known that SSB-Ct interacts with the χ subunit of the CLC, which is important for its remodeling and for stimulation of its β_2_ loading activity (Newcomb et al. 2022) that in turn involves a handover of the primed DNA from the DnaG primase, which also interacts with SSB during primer synthesis (Yuzhakov et al. 1999). However, additional interactions of SSB with Pol IIIα have been identified (Bianco 2021, McIsaac 2022), and it is tempting to speculate that one of their roles might be to facilitate the displacement of SSB during Okazaki fragment synthesis (Sokoloski et al. 2016). Additionally, the formation of the RecA nucleoprotein filament, necessary for SOS activation, also requires SSB displacement, which is usually facilitated by other protein complexes such as RecFOR. Both RecA and RecO interact with SSB (Hobbs et al. 2007, Wu et al. 2017).

SSB mediates protein–protein interactions via its indispensable C-terminal region as well as the IDL, and besides the ones mentioned above, its interactome encompasses at least another 15 proteins, including SSB itself (Bianco et al. 2017, Bianco 2021). How these proteins are important for DNA replication and how they are affected by the interaction with SSB has been extensively reviewed (Shereda et al. 2008). Here, instead, we will focus on our understanding of SSB dynamics, which was enabled by novel research. In relation to that, another crucial role of SSB stems from its chemical properties, namely, the propensity to aggregate. It has been shown that SSB tends to form condensates *in vitro* via a process called liquid–liquid phase separation (LLPS) that is driven by its IDL and SSB-Ct that form multiple weak contacts with neighboring SSB tetramers (Harami et al. 2020). These condensates can store a significant amount of proteins, concentrating them at the sites of DNA replication.

A recently developed super-resolution imaging system optimized for use with prokaryotic cells offers a glimpse into SSB dynamics in a living *E. coli* (Zhao et al. 2019). Under unperturbed conditions, SSB forms multiple foci within the cell with particular enrichment at the inner cell membrane, where it binds phospholipids. However, the situation changes upon DNA damage as under these conditions, SSB tends to form distinct spots along the genome, distally from the membrane (Zhao et al. 2019). Formation of the aforementioned liquid condensates in these spots is a likely explanation for this observation (see the section “Discussion” in Harami et al. 2020). Accordingly, it stands to reason that SSB would be attracted, for example, to the stalled replication forks by the exposed ssDNA, where these replisome-proximal SSB condensates would deliver different proteins to the sites of DNA damage, facilitating quick damage repair and/or tolerance. Indeed, in recent live-cell imaging studies, under conditions of replication stress, DNA Pol IV, RecG, and PriA were shown to be enriched near the replication fork, and at least in the case of Pol IV, it is dependent on the interaction with SSB (Chang et al. 2022, Thrall et al. 2022). Hence, it can be said that SSB plays a role in the regulation of the DNA damage response, not only by controlling access of RecA to DNA, but also by mobilizing DNA repair and damage tolerance factors that facilitate, e.g. HR, replication restart after fork collapse, direct damage repair, TLS (Shereda et al. 2008), and possibly bypass of leading-strand–template DNA gaps (Stanage et al. 2017). These factors might be delivered not only to the sites of ongoing replication but also others, as the damage may occur randomly across the genome.

Although LLPS is inhibited by ssDNA, some SSB condensates might also form at the replication fork under physiological conditions (Harami et al. 2020). Thus, normally, SSB probably still plays a role in enriching certain factors near the fork, for example, RNase HI (Fig. 1A) (Petzold et al. 2015, Wolak et al. 2020). It is also worth mentioning that SSB sequestration at the cell membrane is reminiscent of the mechanism of UmuC activation delay during SOS induction (Robinson et al. 2015). Thus, one can speculate that SSB might also play a role in preventing access of certain DNA repair/damage tolerance proteins under normal conditions by keeping them away from the fork. If this were true, it might be interesting in the future to understand how the compartmentation of specific proteins is achieved, as for example, in untreated cells, there is a strong enrichment of RNase HI at the fork (Wolak et al. 2020), but much less so in case of Pol IV (Thrall et al. 2017). (The caveat here is that RNase HI colocalization with the β_2_ clamp, but Pol IV with SSB, was assayed in the respective studies).

## Factors influencing the fidelity of DNA replication

Although the three major factors influencing the base fidelity of DNA replication have been known since the 1990s, the development of novel methods such as Cryo-EM and live cell imaging, as well as the popularization of deep sequencing techniques, enabled a better insight into their behavior in living cells as well as the structural intricacies involved. Subsequently, some models of how they are triggered, how they act, and what their specificity is had to be revised. New players in the overall DNA replication fidelity were also identified, with the most prominent example being the abundance of ribonucleotides in DNA.

### Base fidelity

#### Nucleotide selection

The spatial considerations involved in the selection of nucleotides with the correct base are well-understood; they are common for all DNA polymerases and have been well-described (Kunkel and Bebenek 2000, Ludmann and Marx 2016). In brief, DNA polymerases select nucleotides according to the rules of Watson–Crick pairing, which are enforced by the shape of the active site. Correct pairing ensures that the size of the pair, dictated by the size of the bases and the length of hydrogen bonds, falls within the spatial constraints of the active site. Other important factors are minor groove scanning, i.e. hydrogen bond formation between the nitrogenous bases and the active site residues, as well as base stacking (Ludmann and Marx 2016). However, genetic studies reveal that Pol III mutations leading to a mutator phenotype are sometimes located in amino acids that do not make direct contact with DNA or the incoming nucleotide. For example, mutations in Pol III Serine 759 are thought to cause impaired closing of the fingers domain over the palm domain during catalysis, which might contribute to increased mutagenesis observed *in vivo* for example due to improper geometry of the active site in the closed conformation (Parasuram et al. 2018, Vaisman et al. 2021). Apart from that, the propensity of DNA polymerases to mispair deoxyribonucleotides also depends on the sequence context and their capability to extend the mismatch. For example, in strains deficient in both proofreading and MMR, transitions are much more frequent than transversions, and they are more likely to occur at the 5′N**A/G**C3′+5′G**T/C**N3′ sites (Lee et al. 2012, Niccum et al. 2018). Additionally, template or primer misalignment are common sources of insertions and deletions (Kunkel and Bebenek 2000, Niccum et al. 2018).

#### Intrinsic proofreading

The textbook view of the transition from DNA synthesis to proofreading upon mismatch creation is that the mispaired nucleotide induces structural changes that lead to the primer terminus being passed from the polymerase to the exonuclease. These were thought to be mediated by the movement of Pol IIIε due to the presence of the flexible linker. However, multiple lines of evidence support the hypothesis that, at least in the case of Pol III, the exonucleolytic activity is regulated thermodynamically. First, using the single-molecule optical tweezers approach, it has been shown that the polymerase and the exonuclease activities are independent. The polymerase preferentially binds the primer–template junction, whereas the exonuclease preferentially binds ssDNA and can similarly cleave mismatched primers as well as free ssDNA. While the rate of initiation by the polymerase does not depend on force, the rate of initiation by the exonuclease is force-dependent (Naufer et al. 2017). These results indicated that it is the instability of the mismatched primer rather than duplex distortion that initiates proofreading because the polymerase preferably binds a stable primer. Second, although the distance between the polymerase and the exonuclease active site is greater than 7 nm (Ozawa et al. 2013), a Cryo-EM structure of the Pol III core in proofreading mode revealed that when Pol III switches to proofreading, the core undergoes very little structural changes, with a small movement of the thumb domain away from DNA, a shift of the exonuclease toward DNA, and DNA itself anchored to the internal surface of the β_2_ (Fernandez-Leiro et al. 2017). Using FRET, it has been shown that the time required to switch from polymerization to exonucleolysis does not depend on the strength of the β–ε interaction, suggesting that it is not broken during switch (Park et al. 2018). Taken together, these data corroborate the model that the primer instability drives proofreading, and molecular dynamics simulations supported by *in vitro* biochemistry offer a glimpse into how its journey to the exonuclease is guided by the fine motions of the Pol III core (Dodd et al. 2020). Interestingly, the mismatches leading to transversion mutations are repaired by proofreading more efficiently than those resulting in transitions (for review, see Bębenek and Ziuzia-Graczyk 2018).

#### Polymerase exchange and extrinsic proofreading: differential fidelity of the leading and lagging DNA strands

DNA polymerase exchange at the replication fork might have a profound impact on the fidelity of DNA replication. It is known that in wild-type *E. coli*, the lagging DNA strand is replicated with a higher fidelity than the leading strand (Fijalkowska et al. 1998, Lee et al. 2012). The differences have been ascribed to the frequent dissociation of the replicase from the terminal mismatch during DNA synthesis (Fig. 4B). As the lagging DNA strand is replicated discontinuously, the dissociation events are assumed to be more frequent on this strand. Upon dissociation, reassociation of an exonuclease-proficient DNA polymerase via its exonuclease activity (i.e. a proofreader such as Pol IIIε or Pol II exo) would likely result in removing the mismatch (Banach-Orlowska et al. 2005), contributing to the high fidelity of lagging-strand synthesis. Conversely, the binding of a low-fidelity, proofreading-deficient DNA polymerase such as Pol IV or Pol V would result in the extension of the mismatch (Fig. 4B), leading to strand-bias reversion and the lagging strand being more mutagenic (Maliszewska-Tkaczyk et al. 2000, Kuban et al. 2004, 2005). The latter phenomenon has been called “spontaneous mutator activity” or “untargeted SOS mutagenesis” to distinguish it from the DNA damage-induced mutator activity, which is not strand-biased as it is dependent on the presence of DNA damage, which can occur on both template strands (Gawel et al. 2002).

The model has been confirmed in further experiments utilizing the Pol IIIα “antimutator” alleles (such as *dnaE915*) with an increased rate of dissociation from DNA (Maslowska et al. 2018, Makiela-Dzbenska et al. 2019). One might expect that an increased chance of replicase dissociation should have little effect on the lagging-strand replication fidelity as this strand is normally replicated in a discontinuous manner. However, it could influence the mutation rates on the leading strand because polymerase exchange now becomes an important replication fidelity factor on this strand as well. Thus, in strains expressing the “antimutator” alleles, due to the more frequent dissociation of Pol III from the mispair, an antimutator effect has been observed compared to the wild-type strain because the proofreading-proficient Pol IIIε and Pol II can now more efficiently remove terminal mismatches not only from the lagging but also from the leading DNA strand. Consistent with the increased access of low-fidelity proofreading-deficient DNA polymerases to DNA replication, overproduction of Pol IV or constitutive activation of Pol V in *dnaE915* strains resulted in a mutator phenotype, which was then observed for both leading and lagging DNA strands (Maslowska et al. 2018, Makiela-Dzbenska et al. 2019). These findings were recapitulated by other laboratories: preferential access of Pol IV to the lagging-strand replication has been observed *in vitro* (Yuan et al. 2016), and whole-genome sequencing approaches have shown that Pol V preferentially replicates the lagging strand in constitutively SOS-induced strains (Niccum et al. 2018, Faraz et al. 2021).

#### MMR system

The last line of defense against mismatched nucleotides is the MMR system. The crude model of *E. coli* MMR comprising MutS, MutL, MutH, UvrD, SSB, an exonuclease, a DNA polymerase, and a ligase is well-established, but has been expanded and revised owing to more recent single-molecule and Cryo-EM studies. MMR is initiated by MutS_2_, which forms a circular dimer responsible for scanning the DNA for mismatch-induced conformation disruptions (or indel-producing looped-out nucleotides). The presence of a mismatch induces conformational changes that make MutS_2_ competent for binding MutL (Fernandez-Leiro et al. 2017). ATP binding allows it to act as a clamp loader and recruit the dimeric MutL_2_ clamp (Yang et al. 2022). Both can move bidirectionally on the DNA (Hasan and Leach 2015). The current model is that the MutS_2_ clamp does not stay at the mismatch site but diffuses, and thus, multiple MutS_2_ dimers can be engaged by a single mismatch (Hao et al. 2020). Additionally, live-cell imaging revealed that MutL_2_ is more abundant at the mismatch site than MutS_2_, suggesting that multiple MutL_2_ dimers are loaded per repair event (Elez et al. 2012). This finding is in line with recent evidence showing that MutS_2_–MutL_2_ interaction is dynamic and that MutS_2_ is not required for MutH activity, suggesting that its primary role is to load MutL_2_ and arguing against the general consensus that MutS_2_ and MutL_2_ form a stable complex (Yang et al. 2022).

MutL_2_ does, however, recruit and form a searching complex with the MutH restriction endonuclease, which cleaves the unmethylated strand at the 5′ side of the recognized GATC sites (Liu et al. 2016). Near the cleavage site, MutL_2_ captures the UvrD helicase that unwinds DNA 3′→5′, and thus exposes the template for resynthesis. The general consensus was that the ssDNA fragment displaced by the helicase is cleaved by one of the cellular exonucleases, but the recent single-molecule biochemical study suggests that this is not necessarily the case (Liu et al. 2019). The exposed gapped fragment of the chromosome is covered by SSB, and then DNA Pol III is engaged to resynthesize the DNA patch. The size of the patch is governed by the distance between GATC sites and can be as big as 1 kb. An additional role of MutL_2_ is protecting the 3′ end of the resected daughter strand from the premature activity of Pol III (Borsellini et al. 2022).

Despite these advances, the model of methyl-directed MMR is far from complete, and important questions remain to be answered. First, it is not entirely clear how MMR is recruited to DNA. It is known that both MutL_2_ and MutS_2_ interact with the β_2_ clamp, with MutS containing two clamp-binding sites in its N- and C-terminal domains (López De Saro et al. 2006). Disruption of the C-terminal motif, which confers strong interaction, does not affect repair, but mutations in the weaker N-terminal-binding site impair MMR activity. Based upon these observations, it has been initially proposed that when Pol III dissociates from the clamp, MutS_2_ binds and scans for mismatches directly behind the fork (López De Saro et al. 2006). However, as β_2_ clamps are more abundant on the lagging strand, this model would imply that MMR might be more efficient on one strand than on the other, for what there is no supporting genetic evidence (Niccum et al. 2018). Additionally, further biochemical studies suggested that mutations in the N-terminal motif result in less stable protein, which is the likely cause of the hypermutator phenotype (Pluciennik et al. 2009). For the same reason, an alternative model suggesting that replisome-bound clamps serve as launching pads for MutS_2_ also seems unlikely (Hasan and Leach 2015). One explanation is that there are no specific recruiters but given that MMR activity hinges on MutH cleaving a mismatch-proximal GATC site before it is methylated by Dam, and Dam action is normally delayed by SeqA, one might entertain the idea that Dam and/or SeqA could contribute to MMR deposition. Indeed, there are some data from bacterial 2-hybrid system suggesting that Dam and MMR proteins interact *in vivo* (Tsai 2019). Another important problem is how the directionality of DNA unwinding is achieved, assuming that UvrD can only translocate 3′→5′, but MutH moves bidirectionally after being loaded by MutS_2_. It stands to reason that there must be a signal that precludes UvrD translocation when it is bound 5′ to the mismatch, as in this case, DNA would be unproductively unwound away from the mismatch. At last, it is unknown how the substrate is handed over to the DNA polymerase for resynthesis. As the gap might be quite big, it is generally thought that the polymerase is assisted by the processivity clamp. Thus, β_2_ could be a likely suspect as it interacts with both Pol III and MutS and MutL. However, as argued before, MutS_2_ interaction with β_2_ is not important for this activity, and disrupting the MutL_2_–β_2_ interaction results in only a mild mutator phenotype, suggesting that β_2_ clamp is not important for substrate handover (Pillon et al. 2015).

In contrast to proofreading, *E. coli* MMR mainly repairs transitions rather than transversions. Correct base pairing, proofreading, and MMR ensure the high fidelity of DNA replication at one mutation per ∼10^10^ paired bases or per ∼2 × 10^3^ replication cycles (Schaaper 1993, Lee et al. 2012). It is worth mentioning that MMR’s capacity to repair replication errors is limited, and when Pol III’s proofreading activity is severely impaired, MMR might easily become overwhelmed (Fijalkowska and Schaaper 1996, Niccum et al. 2018).

#### DNA damage

DNA damage is an important source of genetic instability. The sources of DNA damage can be grouped into endogenous (such as oxidative stress) and exogenous (e.g. UV irradiation, exposure to alkylating agents or antibiotics). Genetic studies utilizing mutation accumulation (MA) assays in strains lacking major DNA repair or damage tolerance pathways reveal that when cells are not exposed to exogenous stress, the only major source of mutations is oxidative stress, leading to the formation of 8-oxo-G (Foster et al. 2015, Bhawsinghka et al. 2023), with a minor effect of spontaneous cytosine deamination (Bhagwat et al. 2016).

Exogeneous damage is frequently mutagenic as it might lead to activation of the TLS polymerases (Robinson et al. 2015, Henrikus et al. 2018). TLS is not a repair mechanism but rather a tolerance mechanism, as the lesion is not removed but bypassed at the cost of fidelity. Thus, instead, cells usually attempt to suppress TLS by engaging other pathways that are normally error-free (e.g. NER or HR) (Naiman et al. 2016). In contrast to the spontaneous mutator phenotype, damage-induced mutagenesis is not strand-biased, as lesions might occur on both DNA strands (Gawel et al. 2002). Recently, a mechanism of how cells transiently elevate their mutation rates to facilitate the emergence of antibiotic resistance in *E. coli* has been described (Gutierrez et al. 2013, Pribis et al. 2019, Zhai et al. 2023). Exposure to a subinhibitory concentration of ciprofloxacin, a DSB-inducing agent, launches a cascade of signaling that leads to the consecutive activation of the RecA-dependent SOS response, the ppGpp-dependent stringent response, and the RpoS-dependent general stress response. Interestingly, this elaborate network of signaling is induced only in a subset (∼20%) of cells that show elevated levels of reactive oxygen species (ROS) after SOS induction, and this subpopulation of cells exhibits a hypermutator phenotype (400 times over the remaining cells) (Pribis et al. 2019, Zhai et al. 2023). Thus, while other cells remain stable, these “gambler” cells undertake the risk of the stress phenotype to help develop antibiotic resistance. The role of ROS in this process is in line with the previously described mutator effect of inactivation of oxidative damage repair (Foster et al. 2015) and has been lately receiving more appreciation (Qi et al. 2023). Importantly, as other work shows that ppGpp binding to the RNAP might promote its backtracking (Kamarthapu et al. 2016), the proposed model suggests that mutagenic repair might be concentrated at the sites of heavy transcription, possibly driving the evolution of strongly expressed genes (see the section “Discussion” in Zhai et al. 2023).

#### The randomness and the nonrandomness of genomic mutations

The above-described model of how cells risk a mutator phenotype to adapt to harsh environmental conditions raises the question of whether the mutagenesis observed in living cells is truly random. There are many facets to this problem, and much insight was provided from MA assays together with WGS analyses, as those studies look at mutations in living cells at a genome-wide scale. From the analysis of the rates of mutations in coding versus noncoding regions, synonymous versus nonsynonymous mutations, codon usage, the rate of terminating mutations, and the rate of deleterious mutations, it has been concluded there is little selective pressure apart from slight bias toward noncoding regions in the DNA of wild-type *E. coli* (Lee et al. 2012). However, the data gathered from MMR- or exo-strains hinted at the possibility of there being a selective pressure to acquire mutator phenotype suppressors (Niccum et al. 2018). Whether this might be evidence for nonrandomness of mutations depends on the definition of randomness in this context, as one might not necessarily expect that mutations in MMR- or exo-strains would be concentrated at the genomic regions containing genes where such suppressors are more likely to occur. This is hard to investigate, but mutations were 20% more likely to occur in coding genes in MMR- or exo-strains (Niccum et al. 2018).

It is worth mentioning that MA assays reveal significant differences in mutation rates across different sequence contexts, which might reflect differential propensities of particular DNA polymerases to create and extend mismatches and/or indels, which were documented in the past in various *in vitro* studies, but also possibly preferences of the repair mechanisms as well (Lee et al. 2012, Niccum et al. 2018, 2020). There is also a growing body of evidence that in certain bacteria, replication–transcription conflicts might be a significant source of genome instability (Lang and Merrikh 2018). The studies performed in *E. coli* argue against this, as there was little correlation between mutation rates and the level of transcription, gene orientation with respect to replication, or the transcribed and the nontranscribed strand (Lee et al. 2012, Foster et al. 2021). Thus, it seems that unlike in many pathogenic bacteria, TC repair is not a major source of genome instability in *E. coli* (Foster et al. 2021).

There is, however, an interesting observation that in *E. coli* and some other prokaryotes, mutation rates across the chromosome form a wave-like pattern symmetrical around the origin of replication (Niccum et al. 2019). One important cause of this pattern is fluctuations of the dNTP pools during DNA synthesis, as changes in dNTP concentrations are known to affect the rate of synthesis and, thus, the chance for error correction. Indeed, there is a body of evidence showing that perturbances in dNTP pools have a significant impact on replication fidelity (Ahluwalia and Schaaper 2013, Schaaper and Mathews 2013, Gawel et al. 2014, Maslowska et al. 2015, Tse et al. 2016). Mutation rate distribution was also changed upon loss of nucleoid-binding proteins HU and Fis, indicating that the fidelity is compromised when the chromosome is highly structured. Another source of genome instability seems to be replication fork pausing or stalling, as Rep deficiency disrupted the pattern (Niccum et al. 2019).

### Sugar fidelity

#### Ribonucleotide incorporation into DNA by the replicase

Apart from correctly pairing the nitrogenous bases, during DNA synthesis, DNA polymerases face the equally important task of selecting nucleotides with the right sugar (Joyce 1997, Brown and Suo 2011), complicated by the fact that the cellular concentrations of the ribonucleotides can exceed those of the corresponding deoxyribonucleotides over a 100-fold (Bennett et al. 2009, Ferraro et al. 2009, Nick McElhinny et al. 2010, Cerritelli and Crouch 2016). It was only in the 2010s that the extent of ribonucleotide incorporation during DNA replication was fully appreciated. It is now known that ribonucleotides are the most common noncanonical nucleotides in DNA and are three orders of magnitude more frequent than mismatches (Nick McElhinny et al. 2010, Yao et al. 2013, Vaisman and Woodgate 2015). In *E. coli*, anywhere between 200 and 600 ribonucleotides are incorporated during a single replication cycle (Cronan et al. 2019, Zatopek et al. 2019).

In most DNA polymerases, sugar selection relies on a single amino acid residue termed the “steric gate”. The steric gate is a bulky amino acid whose side chain localizes in the vicinity of the 2′ carbon of the nucleotide’s sugar moiety, creating a steric hindrance whenever a ribonucleotide positions itself at the active site (Joyce 1997, Brown and Suo 2011). For example, in *E. coli* Pol III, the steric gate is His760 (Fig. 3C) (Parasuram et al. 2018). Other bulky amino acids such as tyrosine, phenylalanine, and glutamic acid are frequently used as steric gates (Joyce 1997, Brown and Suo 2011). Mutating the steric gate of a replicase usually results in a catalytically dead variant, whereas the steric gate mutants of other polymerases, such as the TLS polymerases, exhibit significantly increased ribonucleotide incorporation rates due to the very low sugar selectivity. Some DNA polymerases, but seemingly not in *E. coli*, rely on a “steric fence” formed by the protein backbone for ribose discrimination (Brown et al. 2010, Cavanaugh et al. 2010, 2011). Additionally, it has been shown that apart from the steric gate, *E. coli* Pol IV also has a polar filter residue that draws the 2′-OH of the ribonucleotide close to the protein surface, creating a clash (Johnson et al. 2019). Ribonucleotide incorporation rates vary significantly among DNA polymerases, with the replicases usually exhibiting higher sugar discrimination. *E. coli* Pol III incorporates roughly one rNMP per 2300 nucleotides *in vitro* (Yao et al. 2013, Schroeder et al. 2015). DNA Pol IV has a rather high sugar selectivity, comparable to that of Pol III, while Pol V shows poor sugar discrimination (Vaisman et al. 2012).

Another significant source of ribonucleotides in DNA, although transiently, is primer synthesis by primases. Primers constitute roughly ∼1% of the lagging strand and, in *E. coli*, are removed via a Pol I- and RNase HI-dependent pathway(s) described earlier.

Notably, RNA transcripts may occasionally invade DNA behind the RNAP, and if not removed, they can prime the DNA synthesis (Pomerantz and O’Donnell 2008). This is especially true in bacteria where replication and transcription are not temporally separated, and replisomes are likely to encounter transcription machinery. As already mentioned, in some bacteria, such as *Bacillus subtilis* or *Salmonella typhimurium*, but not in *E. coli*, replication–transcription conflicts are responsible for the hypermutator phenotype and contribute to the development of antibiotic resistance (Lang and Merrikh 2018). However, sometimes, after transcription, the RNA transcripts do not disengage from DNA, forming so-called R-loops. R-loops are naturally used for the initiation of replication of ColE1-type plasmids (Naito and Uchida 1986, Subia and Kogoma 1986), but overall, their presence in genomic DNA has deleterious consequences. In bacteria, they can initiate replication from noncanonical origin sites, leading to constitutive stable DNA replication (cSDR) (Asai and Kogoma 1994, Kogoma 1997). cSDR is initiated at the heavily transcribed regions of DNA such as *rrn* (encoding rRNAs) and significantly changes the replication profile in *E. coli* (Maduike et al. 2014, Dimude et al. 2015). cSDR is *oriC*-independent and strong enough to maintain DNA synthesis in the absence of DnaA. Uncontrolled replication in both directions would lead to frequent fork collapse, potentially creating multiple single-stranded regions prone to DSBs. Another source of DSBs is the creation of R-tracts when R-loops are incorporated into DNA as primers (Kouzminova et al. 2017). Additionally, R-loops may cause replication fork stalling and, if not displaced, require replication restart above damage in bacteria (Kouzminova and Kuzminov 2021). Many proteins are involved in R-loop repair, most notably RNase HI. The repair and significance of R-loops in bacteria and eukaryotes were extensively reviewed in Brickner et al. (2022) and McLean et al. (2022).

#### RNase HI

**Figure 6.**
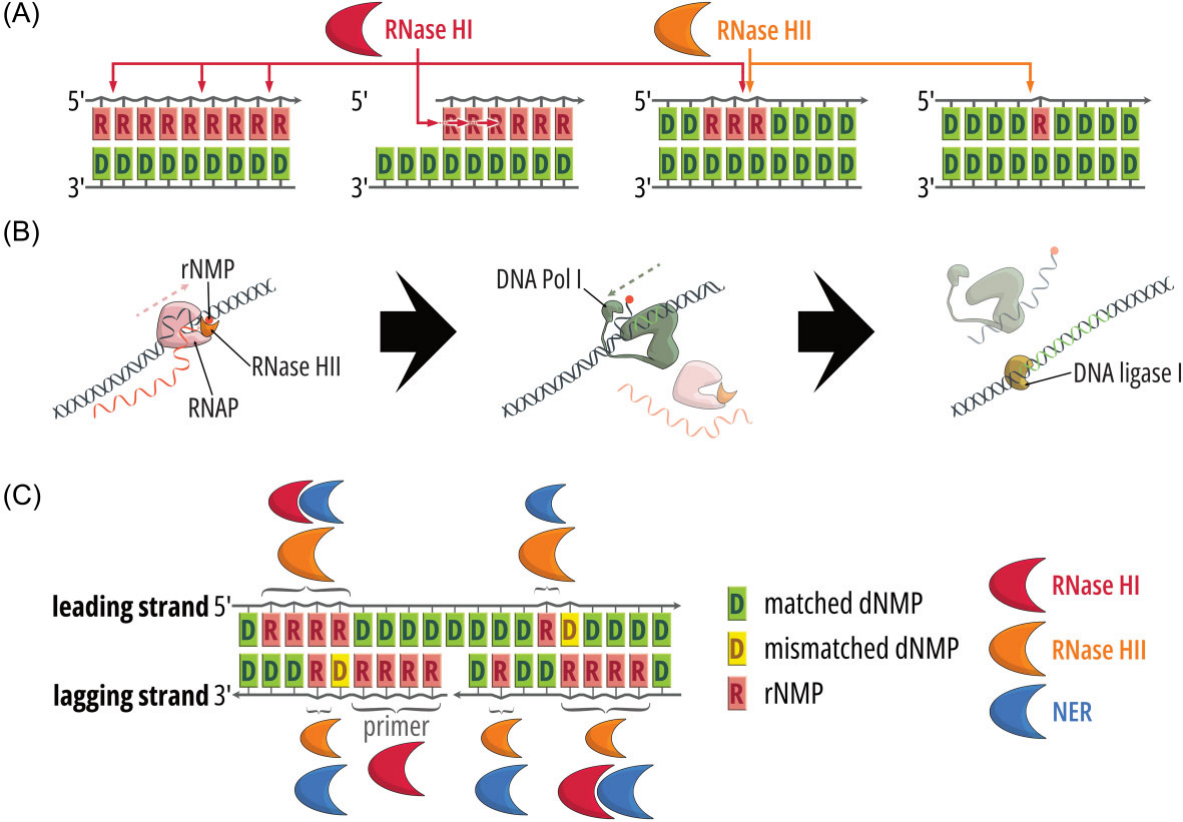
Ribonucleotide excision repair (RER) in *E. coli*. (A) Substrate specificity of RNases HI and HII. RNase HI recognizes polyribonucleotide (3+ nt) tracts, or RNA strands hybridized to DNA. The cleavage site is at least two ribonucleotides from the 5′ end of the tract. RNase HI cleaves the RNA patch distributively, producing a wide range of products of differing lengths. Additionally, *E. coli* RNase HI was shown to work as a processive exoribonuclease in the presence of a 3′ overhang in the opposite strand, i.e. on a substrate mimicking an Okazaki primer. In contrast, RNase HII recognizes single ribonucleotides in a DNA strand. This enzyme is a junction endoribonuclease that preferentially cleaves at the 5′ side of the RNA–DNA junction. (B) Model of *E. coli* transcription-coupled RER. RNase HII rides in front of the RNAP, scanning the transcribed strand for ribonucleotides. Cleavage of the template strand at the 5′ side of the ribonucleotide probably leads to transcription termination and RNAP dissociation, upon which DNA polymerase I resynthesizes a fragment of DNA. The flap is removed by Pol I’s innate flap endonuclease (FEN) activity. Lastly, DNA ligase I removes the remaining nick. (C) Strand specificity of RER in *E. coli*. RNase HII-dependent RER is the primary pathway of ribonucleotide removal with a particularly important role on the leading strand. In contrast, on the lagging strand, it cooperates with other RER pathways that are dependent on the activities of RNase HI and NER. Additionally, RNase HI stimulates the repair of single ribonucleotides on the lagging strand, and the possible mechanism involves RNase HI participation in Okazaki fragment maturation (more details in text). Notably, under certain conditions RER may stimulate the repair of mismatched deoxyribonucleotides, contributing to the high fidelity of DNA replication.

Hybrid ribonucleases (RNases H) are nonsequence-specific endoribonucleases that recognize and cleave RNA parts in the RNA:DNA hybrids (Cerritelli and Crouch 2009, Tadokoro et al. 2009, Hyjek et al. 2019). They belong to the RNase H-like superfamily that also comprises HIV-1 reverse transcriptase, transposases, HJ resolvases, and other nucleases (Majorek et al. 2014). RNase HI encoded by the *rnhA* gene is responsible for cleaving RNA strands in the RNA:DNA hybrids (or hybrids of a DNA strand and a chimeric strand that contains DNA and RNA fragments). It is a single-subunit protein with a catalytic domain that preferentially binds the RNA:DNA hybrids, and this preference is achieved thanks to (a) the interactions of four 2′-OH groups of the RNA strand with the protein chain and (b) a forced conformational change of the DNA strand sugar puckers to a B form, unfavorable for RNA (Hyjek et al. 2019). Because of this binding mode, it has been proposed that RNase HI requires at least four consecutive ribonucleotides in the RNA fragment; however, cleavage of a chimeric DNA strand containing a patch of three ribonucleotides has been reported (Haruki et al. 2002, Reijns et al. 2012). Therefore, it seems that at least two ribonucleotides are required at the 5′ side and at least one at the 3′ side of the cleavage site for hydrolysis to occur (5′-rN-rN-/-rN-3′, Fig. 6A) (Reijns et al. 2012, Łazowski et al. 2023). Unlike the eukaryotic counterparts, *E. coli* RNase HI (as in most other bacteria) cleaves RNA distributively. Additionally, it has been proposed that on a substrate mimicking an Okazaki primer (RNA patch with a 3′ overhang on the opposite DNA strand), *E. coli* RNase HI can work as a processive exoribonuclease (Fig. 6A) (Lee et al. 2022).

As already mentioned, the primary function of RNase HI is the removal of R-loops. As R-loops were likely used to initiate DNA replication in ancient life, RNase HI played a crucial role in this process, and examples can be seen today. Replication of bacterial plasmids possessing ColE1-type *ori* is initiated from a transcript processed by RNase HI (Naito and Uchida 1986, Subia and Kogoma 1986). RNase HI was also reported to be required for the completion of replication of *E. coli* DNA by processing over-replicated genome fragments near the termination site (Wendel et al. 2021). *Escherichia coli* RNase HI interacts with SSB and colocalizes with the replisome, possibly to remove R-loops in front of the replicase (Petzold et al. 2015, Wolak et al. 2020). RNase HI activity may also provide a secondary pathway for primer removal during the Okazaki fragment processing (Ogawa and Okazaki 1984, Balakrishnan and Bambara 2013, Randall et al. 2019, McLean et al. 2022).

#### RNase HII

RNase HII is specialized in identifying single ribonucleotides (Fig. 6A). The specificity of the enzyme is largely dictated by the presence of an absolutely conserved tyrosine residue in the active site. This tyrosine, on the one hand, interacts (along with protein backbone residues) with the 2′-OH of ribose and, on the other hand, positions itself such that the nucleotide located on the 3′ side of the ribonucleotide in the chimeric strand cannot contain a 2′-hydroxyl group due to an imminent steric clash (Hyjek et al. 2019). Hence, RNase HII is a junction ribonuclease that cleaves at the 5′ side of the RNA–DNA junction in the substrate (5′-/-rN-dN-3′, Fig. 6A). Bacterial RNase HII is a monomer (encoded by the *rnhB* gene) and generally requires RNA–DNA junction unless its preferred metal ion Mg^2+^ is swapped with Mn^2+^, in which case RNase HII can cleave distributively like RNase HI (Rychlik et al. 2010).

Bacterial RNase HII does not participate to a great extent in R-loop repair; however, it has been shown in *E. coli* that loss of RNase HII exacerbates growth retardation caused by the lack of RNase HI activity, suggesting some, perhaps secondary, role in this process (Kouzminova et al. 2017). In any case, the primary function of RNase HII is the removal of single ribonucleotides incorporated by DNA polymerases during replication (Schroeder et al. 2015, Vaisman and Woodgate 2015). Loss or impairment of ribonucleotide removal has no phenotypical manifestation in *E. coli*, unlike in eukaryotes (Williams and Kunkel 2022).

#### RER

The term “ribonucleotide excision repair” can be used in a broader sense to describe any pathway engaged in removing single or multiple ribonucleotides from DNA, as evidence shows that more than one exists in both bacteria and eukaryotes (Vaisman and Woodgate 2015, Williams and Kunkel 2022). In general, ribonucleotide repair requires four stages: (a) nucleic acid incision 5′ from the ribonucleotide, (b) resynthesis of DNA, (c) removal of the redundant resynthesized ribonucleotide-containing fragment of the nucleic acid, and (d) ligation of DNA. The canonical RER pathway depends on the activity of RNase HII and was first described in yeast (Sparks et al. 2012). According to the current model of RER in *E. coli*, an incision is followed by SD synthesis by Pol I (Vaisman and Woodgate 2015) (Fig. 6B). Then, Pol I’s innate flap endonuclease activity removes the remaining flap. Alternatively, Pol I has been shown to use its 5′→3′ exonuclease to perform nick-translation synthesis *in vitro* as an alternative mechanism of RER (Vaisman and Woodgate 2015). However, *E. coli* strains expressing Pol I mutants deficient in different activities are proficient in RER, suggesting that Pol III and other cellular exonucleases can replace Pol I during resynthesis and excision steps, respectively (Vaisman et al. 2014).

Unlike in eukaryotic cells, RNase HII does not interact with the β_2_ clamp, and thus, it has been initially assumed that RER occurs passively via diffusion and random binding of RNase HII to DNA. Unexpectedly, it has been shown that *E. coli* RNase HII interacts with the RNAP, making RER a transcription-coupled (TC) repair mechanism similar to NER (Hao et al. 2023). Based on Cryo-EM structures, RNase HII seems to sit in front of the RNAP, actively scanning the transcribed template DNA strand for ribonucleotides, while the polymerase acts as a motor in this context (Fig. 6B). Key to this mechanism is the observation that both sense and antisense strands are actively transcribed in *E. coli* (Hao et al. 2023, Tjaden 2023). Manipulating the level of transcription and disrupting the RNAP–RNase HII interaction interface greatly diminishes RER, although it does not eliminate it, showing that most ribonucleotide repair *in vivo* occurs via TC-RER (Hao et al. 2023).

Perhaps one of the most surprising discoveries in the field was that RER activity might influence the final replication fidelity (in terms of base selection) in *E. coli*. Strains expressing the steric gate mutant of the low fidelity Pol V (Pol V_Y11A) or its orthologue subcloned from an integrative–conjugative element R391 (Pol V_R391__Y13A) exhibit lower mutation rates than the isogenic strains with wild-type polymerases (Vaisman et al. 2012, Walsh et al. 2019). Inactivating RNase HII-dependent RER partially restored the Pol V-dependent mutator phenotype (McDonald et al. 2012, Walsh et al. 2019). This led to the hypothesis that excessive ribonucleotide incorporation and the subsequent enhanced RER activity can lead to the removal of not only ribonucleotides but also adjacent mismatched deoxyribonucleotides during RER-patch resynthesis (McDonald et al. 2012, Vaisman et al. 2012, 2013, 2014, Walsh et al. 2019). Therefore, RER seems to be an important player influencing genetic stability not only by preventing chromosome instability but also by contributing to low mutation rates.

#### NER as the alternative RER pathway

As RNase HII deletion in *E. coli* led to only a partial restoration of the Pol V-dependent mutagenesis in SOS-induced strains expressing Pol V_Y11A, it was theorized that in its absence, backup RER pathways could partially compensate for the lack of RNase HII-RER. This led to the identification of two backup RER pathways in *E. coli* dependent on the activities of RNase HI and NER proteins (McDonald et al. 2012, Vaisman et al. 2013). The role of RNase HI was anticipated as Pol V_Y11A can *in vitro* incorporate polyribonucleotide stretches, a known substrate for RNase HI. However, the involvement of NER was more surprising because the ribonucleotide-induced helix distortion was not expected to be sufficient for UvrAB (DeRose et al. 2012). Based upon the structural analyses and molecular dynamics simulations, it was suggested that the change in electrostatic interactions between the additional 2′ hydroxyl group of the ribose ring and the surface residues of UvrB might contribute to the rifbonucleotide being recognized as a lesion (Cai et al. 2014). Additionally, *in vitro* studies suggest that lesion recognition might be affected by the ribonucleotide being mismatched or by the presence of more ribonucleotides in the vicinity (Vaisman et al. 2013). In contrast, proofreading of ribonucleotides by the replicase, suggested by *in vitro* studies in yeast, seems to make a limited contribution to overall RER in *E. coli* (Łazowski et al. 2023). This is consistent with other observations suggesting that proofreading by Pol IIIε is mostly driven by primer instability, which is probably not the case if the terminal ribonucleotide is correctly paired (see section *Intrinsic proofreading*).

#### Strand specificity of RER

Recently, active site mutants of DNA polymerases characterized by increased ribonucleotide incorporation rates were used together with mutational spectra analyses and WGS-based Hydrolytic Ends sequencing method to study RER efficiency on both DNA strands (Łazowski et al. 2023). Surprisingly, it has been shown that RNase HII activity during RER is more important during leading-strand RER, whereas on the lagging strand, it cooperates with backup RER pathways. This division of labor between the two DNA strands is conserved from normal replication to SOS-induced mutagenesis (Łazowski et al. 2023). One possible explanation for these observations is related to RNase HII involvement in TC-RER. Most heavily transcribed genes are co-oriented with replication, meaning that RNAP is biased toward moving along the leading-strand template (Goehring et al. 2023). Indeed, it seems that the overall transcription level of the leading-strand template is slightly higher than that of the lagging-strand template [personal observations based on the analysis of RNA-seq data in Tjaden (2023)]. Another possibility stems from the more puzzling discovery that RNase HI stimulates the repair of single ribonucleotides on the lagging strand during normal replication despite the lack of such activity *in vitro* (Łazowski et al. 2023). To explain these findings, it has been proposed that RNase HI might be indirectly involved in the repair of primer-proximal single ribonucleotides by virtue of its participation in Okazaki fragment maturation. Although RNase HI is not required for the removal of RNA primers, there is evidence that on a substrate mimicking primed DNA duplex, it might act as a processive exoribonuclease (Lee et al. 2022). As Pol I was shown to resynthesize primers by making an initial nick at the RNA–DNA junction and then replicating up to the nick (Botto et al. 2023), it is possible that shortening the primer disables this mechanism, and thus allows Pol I to resynthesize bigger chunks of DNA, accidentally repairing some ribonucleotides in the process (see the section “Discussion” in Łazowski et al. 2023). That would make Okazaki fragment maturation-associated RER the first, although not the major, mechanism of ribonucleotide repair stimulated by the action of RNase HI, specifically on the lagging strand.

## Concluding remarks

For many years, *E. coli* has proven to be an excellent model organism for studying bacterial physiology, and the results obtained have often become a starting point for the investigations of analogous processes in other bacterial families or eukaryotes. In particular, the mechanisms determining the fidelity of replication, so universal for all organisms, have been extensively studied using *E. coli* as a representative example. This is possible due to the relative simplicity of the *E. coli* replicative apparatus, emphasized by the presence of the single replicative polymerase. For instance, unlike Gram-positive bacteria or eukaryotic cells, where differences in the fidelity of the leading- and lagging-strand replication might be related to the presence of multiple replicases, with *E. coli*, one is able to dismiss this problem and focus on the determinants of replication fidelity (in terms of both base and sugar selection) that stem from the basic principles of DNA replication, such as continuous versus interrupted synthesis of the two DNA strands. This simplicity that we describe is also portrayed in the emerging model of the stochastic nature of the *E. coli* replisome, where, overall, very little control is imposed over its elements. These elements can frequently and freely exchange in the cytosol; moreover, the number of replicative cores or even the active polymerase at the replication fork may change, and some data indicate that replication of the two DNA strands might not be coordinated, in principle allowing for engagement of two separate replicative complexes for DNA replication. Perhaps the most striking example is the observed loss of one daughter chromosome when its replication cannot be finalized due to the presence of a gap in the template, as *E. coli* cells were shown to keep dividing regardless of the gap (Laureti et al. 2015).

At the same time, in certain areas, there is a surprising level of complexity, for example, in the tight regulation of the activity of the most error-prone *E. coli* DNA polymerase, Pol V, and in how different proteins (such as SSB, RNAP, or the helicase) are exploited as either sensors, motors, or mobilizers of DNA repair and damage tolerance factors. Indeed, in this particular problem, it seems that *E. coli* leaves very little to chance, putting to rest the long-standing dispute about macromolecular crowding in the cellular space. The elaborate network of signaling that ultimately leads to the emergence of antibiotic resistance upon DNA damage is a remarkable example of how our understanding of these processes has practical relevance to clinical research.

Despite many years of work, we are still searching for new data that would enable a thorough understanding of the mechanism of DNA replication and the factors determining replication fidelity or controlling the stability of genetic material, and some identified players require more in-depth analysis. Among such issues, there is, for example, the question of how cells achieve timely convergence of the forks if there really is so little coordination between leading- and lagging-strand synthesis, or if, and how, the number of replicative cores at the replication fork is regulated. There are also some open questions that broadly concern the factors ensuring genome stability: the intricacies of MMR recruitment and action remain to be uncovered, as do the mechanisms ensuring timely delivery of the repair and tolerance factors upon DNA damage. If anything, the progress of the last decade, not only regarding research itself but also the availability of novel high-resolution methods, lets one be optimistic.
